# Inflammatory and Nutritional Biomarkers for Overall Survival in Gastric Adenocarcinoma: Comparative Performance and Exploratory Temporal Patterns

**DOI:** 10.3390/diagnostics16213459

**Published:** 2026-10-23

**Authors:** Erdoğan Şeyran, Azer Gökmen, Çağlar Köseoğlu, Emre Hafızoğlu

**Affiliations:** 1 Department of Medical Oncology, Van Training and Research Hospital, University of Health Sciences, Van 65170, Türkiye; 2 Department of Medical Oncology, Gülhane Training and Research Hospital, University of Health Sciences, Ankara 06010, Türkiye; 3 Department of Medical Oncology, Bursa City Hospital, Bursa 16110, Türkiye

**Keywords:** gastric adenocarcinoma, HALP score, inflammation, nutritional status, overall survival, prognostic biomarkers, systemic immune-inflammation index

## Abstract

**Background:** Inflammatory and nutritional biomarkers are associated with survival in gastric cancer; however, their incremental prognostic value beyond routinely available clinical factors and the stability of their associations over time remain uncertain. We compared six routinely available biomarkers within a common clinically adjusted framework and evaluated their associations with overall survival (OS), model performance, temporal variation, and internal robustness. **Methods:** This retrospective single-center cohort study included 255 patients with gastric adenocarcinoma. A prespecified parsimonious Cox model comprising age, sex, Eastern Cooperative Oncology Group performance status, and metastatic disease at diagnosis served as the clinical reference model. The neutrophil-to-lymphocyte ratio (NLR), platelet-to-lymphocyte ratio (PLR), lymphocyte-to-monocyte ratio (LMR), systemic immune-inflammation index (SII), prognostic nutritional index (PNI), and hemoglobin–albumin–lymphocyte–platelet (HALP) score were added separately. Model fit and discrimination, multiple-testing-adjusted associations, time-varying effects, bootstrap internal validation, and influence-diagnostic sensitivity analyses were assessed. **Results:** During follow-up, 227 patients (89.0%) died. Median OS was 11.66 months (95% confidence interval [CI], 9.63–13.47), and reverse Kaplan–Meier median follow-up was 88.51 months (95% CI, 69.72–110.59). After adjustment for the prespecified clinical factors, higher NLR, PLR, and SII were associated with increased mortality, whereas higher LMR, PNI, and HALP were associated with lower mortality; all associations remained significant after Benjamini–Hochberg correction (q ≤ 0.007). The clinical model had a C-index of 0.678. Biomarker addition yielded C-indices ranging from 0.682 to 0.695, with the largest numerical increase observed for PNI (ΔC-index, +0.017). The HALP-extended model yielded the numerically lowest Akaike information criterion. Exploratory time-specific estimates suggested stronger biomarker–mortality associations during early follow-up, although the interaction tests did not remain significant after multiplicity correction. Bootstrap-corrected C-indices were 0.689 for PNI, 0.686 for HALP, and 0.682 for SII, with low optimism and successful convergence of all 1000 resamples. Influence-diagnostic sensitivity analyses preserved the direction and statistical significance of these associations. **Conclusions:** Inflammatory and nutritional biomarkers provided additional prognostic information beyond the prespecified clinical factors. Although all six biomarkers remained associated with OS when evaluated separately, their incremental contribution to overall discrimination was modest. PNI and HALP yielded the most favorable numerical model-performance estimates; however, the absolute differences were modest, and formal paired bootstrap comparisons identified no significant differences between the biomarker-extended models. External and longitudinal validation is required before routine prognostic implementation.

## 1. Introduction

Gastric adenocarcinoma remains a major global health burden and a leading cause of cancer-related mortality, despite advances in diagnostic staging, multimodal treatment, molecular characterization, and biomarker-guided systemic therapy [1,2,3]. Its clinical course is markedly heterogeneous, reflecting differences in anatomical location, disease extent, tumor biology, performance status, treatment eligibility, and host-related factors [2,3,4]. Conventional prognostic assessment relies predominantly on tumor stage and routinely available clinical characteristics; however, these factors do not fully capture the biological interaction between the tumor and the host. There is therefore continued interest in inexpensive, widely available, and readily calculable biomarkers that may complement clinical risk assessment, particularly in real-world settings where comprehensive molecular profiling is not universally available.

Systemic inflammation is increasingly recognized as a central component of cancer progression. Inflammatory signaling may promote angiogenesis, immune escape, tumor-cell survival, invasion, and metastatic dissemination, while the cellular composition of peripheral blood may reflect the balance between tumor-promoting inflammation and antitumor immune competence [5,6]. Neutrophilia and thrombocytosis may indicate an inflammatory and prothrombotic state, whereas lymphopenia may reflect impaired cell-mediated immunity; monocyte-related measures may additionally capture myeloid activation within the host response. These biological relationships form the basis for routinely calculable indices such as the neutrophil-to-lymphocyte ratio (NLR), platelet-to-lymphocyte ratio (PLR), lymphocyte-to-monocyte ratio (LMR), and systemic immune-inflammation index (SII). Previous studies have linked these indices to survival across solid tumors [7], and SII was initially developed as a composite measure integrating circulating neutrophil, platelet, and lymphocyte counts [8]. More recently, pooled evidence specific to gastric cancer has supported an association between elevated SII and adverse survival outcomes [9].

Inflammatory burden represents only one dimension of the host–tumor relationship. Nutritional depletion and hypoalbuminemia are common in patients with cancer and may reflect the interacting effects of reduced dietary intake, tumor-related metabolic alterations, systemic inflammation, and overall disease burden [10]. Composite indices may therefore provide a broader representation of host reserve than any individual laboratory parameter. The prognostic nutritional index (PNI), originally derived from serum albumin and lymphocyte count [11], combines nutritional and immune components, whereas the hemoglobin–albumin–lymphocyte–platelet (HALP) score integrates hematologic, nutritional, inflammatory, and immune features [12]. Recent evidence has supported the prognostic relevance of PNI in gastrectomy cohorts and has examined its performance alongside SII in early gastric cancer [13,14]. Similarly, HALP has been associated with outcomes in curatively treated and metastatic gastric cancer populations [15,16], and a recent meta-analysis confirmed its prognostic relevance across digestive system cancers, including gastric cancer [17]. Evidence from a large European registry has further demonstrated the clinical relevance of PNI in a Western population undergoing multimodal treatment [18].

Despite this growing body of evidence, several uncertainties remain. Many previous studies evaluated a single biomarker, focused on selected surgical or treatment-defined populations, or converted continuous laboratory measures into data-derived categories. Such categorization may result in loss of statistical information, reduce prognostic performance, limit comparability across cohorts, and generate thresholds that are difficult to reproduce in external populations [19]. Furthermore, demonstrating a statistically significant hazard ratio does not necessarily establish that a biomarker meaningfully improves prognostic performance beyond established clinical factors [20]. Comparative evaluations using a common analytical population and the same clinical reference model remain limited, and the temporal stability of biomarker associations has received relatively little attention. This is clinically relevant because a baseline marker may carry substantial prognostic information early after diagnosis while becoming less informative as treatment exposure, disease evolution, and subsequent changes in host status accumulate. When proportional-hazards assumptions are not satisfied, time-varying coefficient models provide a more appropriate representation of such changing associations [21].

Accordingly, this study compared six routinely available inflammatory and nutritional biomarkers—NLR, PLR, LMR, SII, PNI, and HALP—in patients with gastric adenocarcinoma. Each biomarker was evaluated separately against the same prespecified parsimonious clinical reference model to distinguish prognostic association from incremental model performance. We additionally examined whether biomarker associations varied over time and assessed the internal robustness of the leading biomarker-extended models using bootstrap validation and influence-diagnostic sensitivity analyses.

## 2. Materials and Methods

### 2.1. Study Design and Setting

This retrospective, single-center cohort study included adult patients with histologically confirmed gastric adenocarcinoma who were evaluated at the University of Health Sciences, Van Training and Research Hospital, Van, Türkiye, between January 2012 and June 2023. Clinical, pathological, treatment-related, laboratory, and survival data were obtained from institutional electronic medical records and archived patient files. The study was designed to evaluate and compare the prognostic associations of routinely available systemic inflammatory and nutritional biomarkers with overall survival.

The database was reviewed using prespecified eligibility and data-quality criteria before analysis. Follow-up information was available through 10 August 2023. The study was conducted and reported in accordance with the principles of the Declaration of Helsinki and applicable recommendations for retrospective observational studies.

### 2.2. Patient Selection

Patients were eligible if they were aged 18 years or older, had histologically confirmed gastric adenocarcinoma, were diagnosed within the prespecified study period, had sufficient clinical and follow-up information for overall survival assessment, and had pretreatment laboratory measurements available for calculation of all six prespecified biomarkers. Patients were required to have valid baseline data for age, sex, Eastern Cooperative Oncology Group performance status, metastatic disease at diagnosis, hemoglobin, albumin, absolute neutrophil count, absolute lymphocyte count, absolute monocyte count, and platelet count.

Records with a diagnosis outside the prespecified study period, unverifiable core dates, internally inconsistent survival information, or insufficient data for calculation of the prespecified biomarkers were excluded. Patients with non-adenocarcinoma histology were not eligible. Adenocarcinomas arising from the stomach or gastroesophageal junction, including Siewert types II–III, were eligible; Siewert type I tumors were not included. A common complete-case analytical approach was used to ensure that all clinical and biomarker-specific models were fitted in the same population. Of 269 patients assessed for final eligibility, data completeness, and internal consistency, 14 patients (5.2%) were excluded: 13 because of missing or internally discordant data required for the prespecified clinical or biomarker analyses and one because a core survival date could not be reliably verified. The final analytical cohort therefore comprised 255 patients (94.8%). This same cohort was used for the primary Cox regression models, model-performance comparisons, time-varying effect analyses, bootstrap internal validation, influence diagnostics, and Kaplan–Meier analyses.

### 2.3. Data Collection and Clinical Definitions

Demographic, clinical, pathological, treatment-related, laboratory, and survival data were retrospectively extracted from institutional records. The collected clinical variables included age at diagnosis, sex, Eastern Cooperative Oncology Group (ECOG) performance status, metastatic disease at diagnosis, primary tumor location, primary-tumor surgery, date of diagnosis, date of death, and date of last documented follow-up. Receipt of first-line systemic treatment was verified using chemotherapy administration records, treatment orders, and available clinical documentation. Patients without documented treatment at the study center were categorized according to the available clinical documentation as receiving best supportive care, declining treatment, or transferring to another center.

Molecular testing was performed as part of routine clinical care according to clinical indication and test availability. HER2 status was assessed by immunohistochemistry, with confirmatory in situ hybridization for equivocal immunohistochemistry 2+ results when available. PD-L1 combined positive score (CPS) and microsatellite instability/mismatch repair (MSI/MMR) status were recorded when available; however, these assessments were not performed uniformly across the entire cohort. Because of their incomplete availability, these molecular variables were not incorporated into the primary prognostic models.

ECOG performance status was categorized as 0–1 versus ≥ 2 for the survival analyses. Metastatic disease at diagnosis was defined as distant metastatic involvement documented by radiological, pathological, or clinical assessment at the time of initial diagnosis. Clinical stage at diagnosis was retrospectively abstracted from baseline clinical and radiological records and harmonized into broad stage I–IV categories. Patients with distant metastatic disease at diagnosis were classified as stage IV. When the complete stage could not be reconstructed, but the absence of metastatic disease was documented, these patients were retained in the nonmetastatic group and classified as having unavailable complete stage information. Primary tumor location was classified as the stomach or gastroesophageal junction. Gastroesophageal junction tumors were restricted to Siewert types II–III and were managed and recorded as gastric adenocarcinomas within the institutional database. Primary tumor location was summarized descriptively and was not included in the prespecified clinical Cox model because location data were unavailable for a substantial proportion of the cohort. Surgery was not included in the prespecified clinical Cox model because of potential treatment-selection bias and its time-dependent nature relative to the timing of diagnosis and biomarker assessment.

Age was calculated using the verified date of birth and date of diagnosis and was modeled continuously per 10-year increase. The primary clinical adjustment model was prespecified to include age, sex, ECOG performance status, and metastatic disease at diagnosis. This parsimonious model was selected to provide a consistent, clinically interpretable reference model while limiting overfitting and avoiding adjustment for incompletely documented or treatment-dependent variables. Complete tumor stage was not incorporated into the primary clinical model because stage information was unavailable for 32 nonmetastatic patients, whereas metastatic status at diagnosis was available for all 255 patients and provided a uniform indicator of the principal disease-setting distinction. Including complete stage would therefore have reduced the analytical cohort and departed from the common complete-case population used for all primary biomarker comparisons. Nevertheless, metastatic status does not fully capture the prognostic heterogeneity among stages I–III, and residual confounding related to disease stage cannot be excluded. Treatment variables were summarized descriptively and were not incorporated into the primary prognostic models because treatment allocation was nonrandom, depended on disease setting and patient fitness, and could have occurred after the baseline biomarker assessment.

### 2.4. Laboratory Measurements and Data Quality

Baseline laboratory variables included hemoglobin, serum albumin, absolute neutrophil count, absolute lymphocyte count, absolute monocyte count, and platelet count. Baseline measurements were defined as the pretreatment complete blood count and serum albumin values obtained within seven days before the initiation of the first disease-directed treatment. For patients undergoing upfront surgery, the eligible laboratory measurement closest to surgery within the preceding seven days was used. For patients receiving systemic therapy or radiotherapy as their initial treatment, the eligible measurement closest to treatment initiation within the preceding seven days was selected. For patients who did not receive disease-directed treatment, laboratory measurements obtained within seven days of the initial oncological assessment were used. When multiple eligible measurements were available, the measurement closest to the relevant treatment or assessment date was selected. Laboratory values obtained after the initiation of systemic therapy, radiotherapy, surgery, or other disease-directed oncological treatment were not used for biomarker calculation. Accordingly, the calculated biomarkers represented pretreatment systemic inflammatory and nutritional status in the relevant clinical setting.

Complete blood count components were standardized to ×10^9^/L. Hemoglobin and albumin were recorded in g/dL for descriptive presentation. For calculation of the hemoglobin–albumin–lymphocyte–platelet (HALP) score, hemoglobin and albumin were converted to g/L to maintain consistency with the conventional HALP formula.

Before analysis, laboratory values were assessed for unit consistency, implausible values, and internal compatibility with the source variables. Values with potentially discordant units or extreme magnitudes were checked against the available source records. No observation was excluded solely on the basis of being statistically extreme. Patients with missing values for any variable required for the prespecified common complete-case analysis were not included in the analytical cohort. The missing-data mechanism could not be formally established, and no assumption that the excluded data were missing at random was made. Because the excluded records lacked sufficient analyzable information for the prespecified clinical and biomarker variables, a reliable comparison between the excluded patients and the final analytical cohort could not be performed.

### 2.5. Calculation of Inflammatory and Nutritional Biomarkers

All inflammatory and nutritional biomarkers were recalculated from their individual laboratory components. Complete blood count components were standardized to ×10^9^/L before calculation. The following formulas were used:

NLR = absolute neutrophil count/absolute lymphocyte count(1)

PLR = platelet count/absolute lymphocyte count(2)

LMR = absolute lymphocyte count/absolute monocyte count(3)

SII = (platelet count × absolute neutrophil count)/absolute lymphocyte count(4)

PNI = [10 × serum albumin (g/dL)] + [5 × absolute lymphocyte count (×10^9^/L)] (5)

HALP = [hemoglobin (g/L) × serum albumin (g/L) × absolute lymphocyte count (×109/L)]/platelet count (×109/L)(6)

For the calculation of HALP, hemoglobin and albumin values recorded in g/dL were converted to g/L by multiplying each value by 10. The unit-standardized HALP formula was therefore equivalent to the following expression when the original g/dL values were used:

HALP = [100 × hemoglobin (g/dL) × serum albumin (g/dL) × absolute lymphocyte count (×10^9^/L)]/platelet count (×10^9^/L) (7)

All biomarkers were analyzed as continuous variables in the Cox regression analyses. Because NLR, PLR, LMR, SII, and HALP had right-skewed distributions, they were log_2_-transformed before regression modeling. Accordingly, the hazard ratios for these biomarkers represent the relative change in the mortality hazard associated with a two-fold increase in the corresponding biomarker.

PNI was retained on its original continuous scale and divided by five for regression modeling. Its hazard ratio therefore represents the relative change in the mortality hazard associated with a five-unit increase in PNI.

Median-based categorization was used only for descriptive Kaplan–Meier visualization and log-rank comparisons. No outcome-derived cut point or data-driven categorization was used in the primary Cox regression analyses.

### 2.6. Outcome Definition and Follow-Up

The primary outcome was overall survival (OS), defined as the interval from the date of histologically confirmed diagnosis to death from any cause. Patients who were alive at the date of their last documented clinical contact were censored on that date. Survival time was calculated in months.

The database contained follow-up information through 10 August 2023. Median follow-up duration was estimated using the reverse Kaplan–Meier method, in which deaths were treated as censored observations and patients originally censored in the OS analysis were treated as events. Median OS and its 95% confidence interval were estimated using the conventional Kaplan–Meier method.

### 2.7. Statistical Analysis

Statistical analyses were conducted using IBM SPSS Statistics for Windows, version 25.0 (IBM Corp., Armonk, NY, USA), and R software (R Foundation for Statistical Computing, Vienna, Austria; https://www.r-project.org/, accessed on 21 October 2026). Continuous variables were summarized using median and interquartile range because several clinical and laboratory variables showed non-normal or right-skewed distributions. Categorical variables were summarized as frequencies and percentages. Percentages for tumor location and primary-tumor surgery were calculated using the corresponding available-data denominators.

No formal a priori sample-size calculation was performed because of the retrospective design. All patients who met the prespecified eligibility, data-consistency, and complete-case requirements were included. Missing values were not imputed. The final analytical cohort had complete data for the outcome, the four prespecified clinical covariates, and all laboratory components required to calculate the six biomarkers. Therefore, all primary clinical and biomarker-specific regression analyses were performed in the same complete-case cohort of 255 patients. Tumor-location and primary-tumor surgery data were incomplete and were consequently summarized using available-data denominators and were not included in the primary models.

Associations with OS were initially evaluated using univariable Cox proportional hazards regression. Age was modeled continuously per 10-year increase. Sex was modeled as male versus female, ECOG performance status as ≥2 versus 0–1, and metastatic disease at diagnosis as yes versus no. NLR, PLR, LMR, SII, and HALP were entered after log_2_ transformation, and their hazard ratios represent a two-fold increase. PNI was modeled per five-unit increase.

A prespecified multivariable clinical Cox model included age, sex, ECOG performance status, and metastatic disease at diagnosis. Each inflammatory or nutritional biomarker was subsequently added separately to this clinical model. A single model containing all biomarkers simultaneously was not used because the indices share several laboratory components and represent overlapping inflammatory and nutritional constructs. Separate biomarker-extended models allowed the incremental prognostic contribution of each marker to be evaluated against the same clinical reference model while limiting instability related to multicollinearity. To explore whether the individual components of HALP provided independent prognostic information, an exploratory component-level Cox model simultaneously included hemoglobin, serum albumin, absolute lymphocyte count, and platelet count, in addition to age, sex, ECOG performance status, and metastatic disease at diagnosis. Hemoglobin was modeled per 1-g/dL increase, albumin per 0.5-g/dL increase, and lymphocyte and platelet counts after log_2_ transformation, with their hazard ratios representing a two-fold increase. Multicollinearity was evaluated using variance inflation factors (VIFs). Benjamini–Hochberg correction was applied across the four component-specific tests. The component model was compared with the clinical reference model using the likelihood-ratio test, AIC, and Harrell’s C-index. Dimensionality reduction was not performed because the analysis involved only four prespecified and clinically interpretable laboratory variables, and the objective was to estimate their mutually adjusted associations rather than derive a cohort-specific latent component.

Results of the Cox models were reported as hazard ratios (HRs), 95% confidence intervals (CIs), and two-sided *p* values. Model discrimination was quantified using Harrell’s concordance index (C-index). The absolute change in C-index was calculated relative to the prespecified clinical model. Model fit was evaluated using the Akaike information criterion (AIC), with lower values indicating better relative fit. Nested biomarker-extended models were compared with the clinical model using likelihood-ratio tests. Direct comparisons of discrimination among the six biomarker-extended models were performed using 1000 paired bootstrap resamples. Within each bootstrap resample, all six models were refitted using the same resampled patients, and pairwise differences in Harrell C-indices were calculated. Percentile-based 95% confidence intervals and two-sided *p* values were obtained for each paired difference. Benjamini–Hochberg correction was applied across the 15 pairwise model comparisons.

Because six biomarker-specific comparisons were performed, the corresponding biomarker *p* values were adjusted using the Benjamini–Hochberg procedure. Both unadjusted *p* values and Benjamini–Hochberg-adjusted q values were reported. Statistical significance was defined using a two-sided *p* value or q value < 0.05, as applicable.

### 2.8. Assessment of the Proportional Hazards Assumption

The proportional hazards assumption was evaluated using scaled Schoenfeld residuals for individual covariates and global model tests. Because proportionality diagnostics showed nominal evidence of potentially non-constant biomarker effects, additional extended Cox models incorporating biomarker × log-time interaction terms were fitted.

Each time-varying model was adjusted for age, sex, ECOG performance status, and metastatic disease at diagnosis. Time-specific adjusted HRs and 95% CIs were estimated at 3, 6, 12, 24, and 36 months after diagnosis. These estimates were used to characterize whether the prognostic association of each biomarker remained constant or changed during follow-up. When an unadjusted biomarker × log-time interaction *p* value was <0.05, the conventional Cox-model HR was interpreted as a summary association averaged over the observed follow-up period rather than as a constant effect applicable at all time points. The corresponding time-specific HRs were used to explore the potential temporal pattern of the biomarker association. Because temporal variation was evaluated separately for six biomarkers, the biomarker × log-time interaction *p* values were additionally adjusted using the Benjamini–Hochberg procedure. These analyses were considered exploratory, and both unadjusted *p* values and adjusted q values were reported.

### 2.9. Exploratory Effect-Modification Analysis

Because the cohort included both nonmetastatic and metastatic disease, exploratory analyses evaluated whether biomarker associations differed according to metastatic status at diagnosis. For each biomarker, an extended Cox model included age, sex, ECOG performance status, metastatic disease, the biomarker, and a biomarker × metastatic-status interaction. To distinguish disease-setting heterogeneity from temporal variation, additional models jointly incorporated biomarker × log-time, metastatic-status × log-time, and biomarker × metastatic-status × log-time terms. Overall effect modification was evaluated by likelihood-ratio comparison of models with and without the static and time-dependent biomarker × metastatic-status terms. Benjamini–Hochberg correction was applied across the six global effect-modification tests. As a sensitivity analysis, all clinical covariates were additionally permitted to vary with log-time. To provide clinically interpretable stratum-specific estimates, separate exploratory Cox models were additionally fitted within the nonmetastatic and metastatic subgroups. Each model included age, sex, ECOG performance status, and one biomarker; metastatic status was omitted because it was constant within each subgroup. The Benjamini–Hochberg procedure was applied separately across the six biomarker tests within each subgroup. These stratified analyses were considered exploratory, and differences in statistical significance between subgroups were not interpreted as evidence of effect modification; formal between-group heterogeneity was assessed using the interaction models described above.

As an additional sensitivity analysis, receipt of any systemic treatment and primary-tumor surgery were incorporated as time-dependent covariates in each biomarker-extended Cox model. Systemic treatment status was coded as 0 until the earliest recorded initiation date of neoadjuvant, adjuvant, or first-line palliative systemic therapy and as 1 thereafter. Surgery status was similarly coded as 0 until the recorded surgery date and as 1 thereafter. Each model additionally included age, sex, ECOG performance status, metastatic disease at diagnosis, and one biomarker. Benjamini–Hochberg correction was applied across the six biomarker tests. Systemic treatment and surgery were included only as time-dependent adjustment covariates; their coefficients were not interpreted as causal treatment effects.

### 2.10. Internal Validation

Internal validation of model discrimination was performed using 1000 bootstrap resamples. The clinical model and the three leading biomarker-extended models—clinical model plus PNI, clinical model plus HALP, and clinical model plus SII—were evaluated.

For each bootstrap resample, the corresponding model was refitted, and its C-index was calculated both in the bootstrap sample and in the original cohort. Optimism was defined as the difference between bootstrap-sample and original-cohort performance. Mean optimism was subtracted from the apparent C-index to obtain the optimism-corrected C-index.

Bootstrap intervals were defined using the 2.5th and 97.5th percentiles of the optimism-corrected estimates across the successful resamples. All 1000 bootstrap resamples converged successfully.

### 2.11. Influence Diagnostics and Sensitivity Analyses

Influence diagnostics were performed separately for the leading biomarker models containing PNI, HALP, or SII. Influential observations were identified using DFBETAs and a generalized Cook-type measure. An observation was flagged if the maximum absolute DFBETA exceeded 2/√*n* = 0.125 or if the generalized Cook-type measure exceeded 4/*n* = 0.0157.

For each biomarker model, a sensitivity Cox analysis was performed after excluding observations flagged within that specific model. These sensitivity models retained the same clinical adjustment variables as the primary models. Flagged observations were not considered data errors solely because of their statistical influence; the analyses were conducted to determine whether the primary biomarker estimates were disproportionately dependent on a limited number of observations.

### 2.12. Kaplan–Meier Analysis Methods

Kaplan–Meier curves were generated for PNI, HALP, and SII to provide descriptive visualization of survival differences. Patients were categorized using the cohort median value of the corresponding biomarker. The low group included values below the median, whereas the high group included values equal to or above the median.

Survival distributions were compared using the log-rank test. Median OS and 95% CIs were calculated for each group, and numbers at risk were reported at prespecified time points. Median-based groups were used exclusively for graphical and descriptive purposes and were not used in the primary Cox regression models.

### 2.13. Ethical Approval

The study protocol was approved by the Non-Interventional Clinical Research Ethics Committee of the University of Health Sciences, Van Training and Research Hospital (decision no. GOKAEK/2026-04/28; 10 April 2026). The requirement for individual informed consent was waived by the Ethics Committee because of the retrospective design of the study and the use of anonymized routinely collected clinical data. No directly identifiable patient information was included in the analytical dataset.

## 3. Results

### 3.1. Cohort Characteristics and Follow-Up

**Figure 1:**
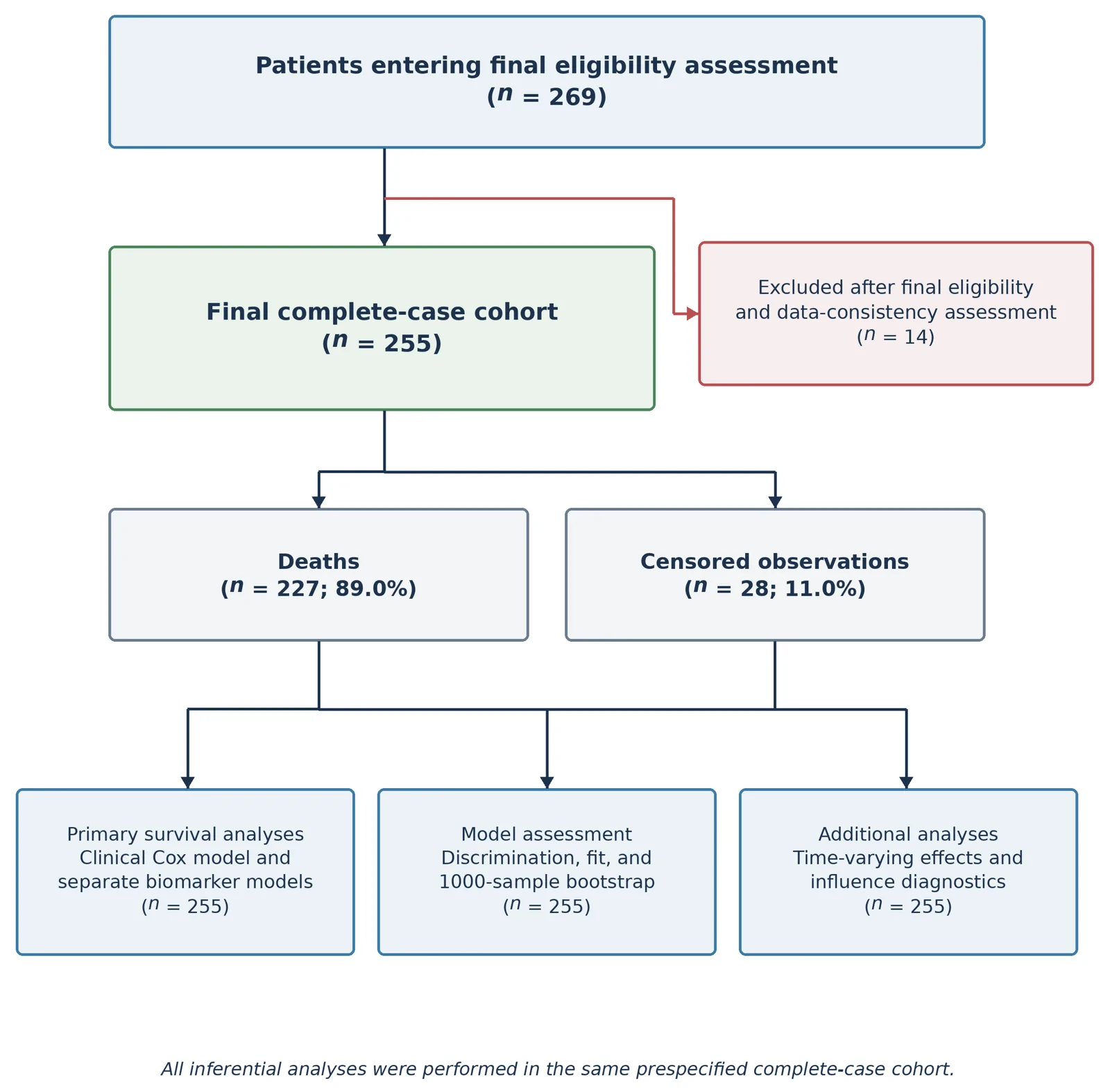
Study flow diagram. Of 269 patients assessed for final eligibility, data completeness, and internal consistency, 14 were excluded because they did not meet the prespecified eligibility, complete-case, or data-consistency requirements. The final analytical cohort comprised 255 patients, including 227 deaths and 28 censored observations. All primary and additional analyses were performed in this common prespecified cohort.

The final complete-case cohort comprised 255 patients with gastric adenocarcinoma (Figure 1). The median age at diagnosis was 63.5 years (interquartile range [IQR], 57.2–71.1 years), and 171 patients (67.1%) were male. Most patients had an ECOG performance status of 0–1 (189 patients, 74.1%), whereas 66 patients (25.9%) had an ECOG performance status of ≥2.

Metastatic disease was present at diagnosis in 117 patients (45.9%), while 138 patients (54.1%) had no documented metastasis at diagnosis. The stage distribution at diagnosis was as follows: stage I in 12 patients (4.7%), stage II in 36 (14.1%), stage III in 58 (22.7%), and stage IV in 117 (45.9%). Complete stage information was unavailable for the remaining 32 nonmetastatic patients (12.5%), although metastatic status was available for all 255 patients. Tumor-location data were available for 188 patients; among these, 135 tumors (71.8%) originated in the stomach, and 53 (28.2%) arose from the gastroesophageal junction. Primary-tumor surgery data were available for 253 patients. Surgery was performed in 112 patients (44.3%) and was not performed in 141 patients (55.7%). All 255 patients had complete data for overall survival, age, sex, ECOG performance status, metastatic disease at diagnosis, and the laboratory components required to calculate NLR, PLR, LMR, SII, PNI, and HALP.

The median baseline hemoglobin concentration was 12.5 g/dL (IQR, 10.5–14.0), and the median serum albumin concentration was 3.80 g/dL (IQR, 3.40–4.13). Median baseline neutrophil, lymphocyte, monocyte, and platelet counts were 4.95 (IQR, 3.92–6.80), 1.64 (IQR, 1.19–2.13), 0.51 (IQR, 0.41–0.71), and 299.0 (IQR, 233.5–363.5) × 10^9^/L, respectively. The median values of the calculated inflammatory and nutritional biomarkers were 3.08 for NLR, 185.5 for PLR, 3.15 for LMR, 928.3 for SII, 46.45 for PNI, and 22.92 for HALP (Table 1).

During follow-up, 227 patients (89.0%) died and 28 patients (11.0%) were censored. The median follow-up duration, estimated using the reverse Kaplan–Meier method, was 88.51 months (95% CI, 69.72–110.59). The median OS for the entire cohort was 11.66 months (95% CI, 9.63–13.47).

### 3.2. Metastatic Disease, Surgery, and Treatment Patterns

Among the 255 patients in the final cohort, 117 (45.9%) had metastatic disease at diagnosis. Primary-tumor surgery was performed in 22 of these patients (18.8%). Among patients without metastatic disease at diagnosis, surgical-status data were available for 136 of 138 patients, of whom 90 (66.2%) underwent surgery.

Among the 117 patients with metastatic disease at diagnosis, 104 (88.9%) received first-line systemic treatment. Eight patients (6.8%) were managed with best supportive care because of poor performance status, and two patients (1.7%) declined systemic treatment. Three patients (2.6%) subsequently presented to another center, and information regarding treatment administered after transfer was unavailable.

Among the 104 patients who received first-line systemic treatment, the chemotherapy backbones were FOLFOX in 57 patients (54.8%), CAPOX in 20 (19.2%), mDCF in 12 (11.5%), FLOT in 5 (4.8%), FOLFIRI in 4 (3.8%), capecitabine monotherapy in 4 (3.8%), and cisplatin plus capecitabine in 2 (1.9%). HER2 results were available for 221 of 255 patients (86.7%), of whom 45 (20.4%) had HER2-positive tumors. Among patients with metastatic disease, HER2 results were available for 107 of 117 patients, and 23 of 107 (21.5%) were HER2-positive. PD-L1 CPS and MSI/MMR results were not sufficiently complete for cohort-level or molecularly stratified analyses. Trastuzumab was administered to 13 patients (12.5% of treated patients), in combination with FOLFOX in 7, CAPOX in 3, capecitabine in 1, and cisplatin plus capecitabine in 2. The trastuzumab-containing regimens were included within the corresponding chemotherapy-backbone categories and were not counted as separate treatment groups. No patient received immune-checkpoint inhibitor therapy during the study period.

Because treatment allocation was nonrandom and strongly influenced by disease characteristics, performance status, patient preference, and clinical fitness, treatment variables were summarized descriptively and were not included in the primary prognostic Cox models.

### 3.3. Univariable and Adjusted Cox Regression Analyses

In univariable Cox regression analyses, older age, impaired performance status, metastatic disease at diagnosis, and all six inflammatory or nutritional biomarkers were significantly associated with OS (Table 2). Each 10-year increase in age was associated with a higher mortality hazard (HR, 1.279; 95% CI, 1.116–1.466; *p* < 0.001). ECOG performance status ≥ 2 was associated with more than a two-fold higher mortality hazard compared with ECOG 0–1 (HR, 2.161; 95% CI, 1.610–2.902; *p* < 0.001). Metastatic disease at diagnosis was also strongly associated with shorter OS (HR, 2.231; 95% CI, 1.704–2.919; *p* < 0.001). Sex was not associated with OS (male vs. female: HR, 0.969; 95% CI, 0.735–1.278; *p* = 0.826).

Higher NLR, PLR, and SII were associated with increased mortality when modeled per two-fold increase. The corresponding HRs were 1.358 (95% CI, 1.183–1.559; *p* < 0.001), 1.557 (95% CI, 1.283–1.891; *p* < 0.001), and 1.458 (95% CI, 1.276–1.666; *p* < 0.001), respectively. In contrast, higher LMR, PNI, and HALP were associated with lower mortality. The HRs were 0.658 (95% CI, 0.559–0.775; *p* < 0.001) for LMR per doubling, 0.779 (95% CI, 0.714–0.850; *p* < 0.001) for PNI per five-unit increase, and 0.665 (95% CI, 0.579–0.763; *p* < 0.001) for HALP per doubling.

**Figure 2:**
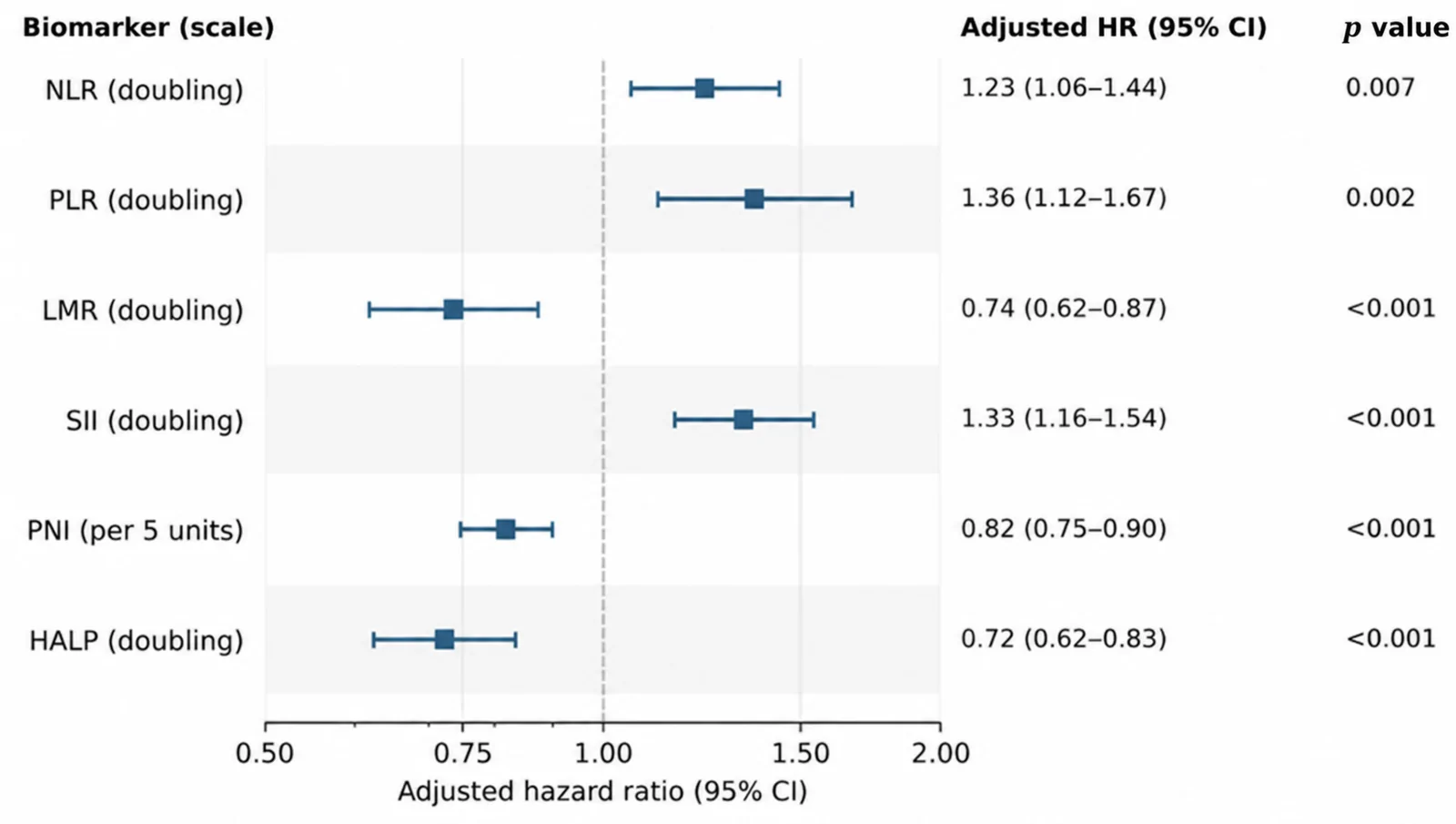
Adjusted associations between inflammatory and nutritional biomarkers and overall survival. Forest plots show adjusted hazard ratios (HRs) and 95% confidence intervals (CIs) derived from separate Cox proportional hazards models. Each biomarker model was adjusted for age, sex, Eastern Cooperative Oncology Group performance status, and metastatic disease at diagnosis. NLR, PLR, LMR, SII, and HALP were log_2_-transformed; their HRs therefore represent a doubling of the corresponding biomarker. The HR for PNI represents a 5-unit increase. The dashed vertical line indicates an HR of 1.00.

The prespecified clinical Cox model included age, sex, ECOG performance status, and metastatic disease at diagnosis. After adjustment for these clinical characteristics, all six biomarkers remained significantly associated with OS when added separately to the same clinical model (Table 3; Figure 2).

For the inflammation-related biomarkers, a two-fold increase in NLR was associated with a higher mortality hazard (adjusted HR, 1.233; 95% CI, 1.059–1.435; *p* = 0.007; BH-adjusted q = 0.007). The corresponding adjusted HRs were 1.365 (95% CI, 1.118–1.666; *p* = 0.002; q = 0.003) for PLR and 1.335 (95% CI, 1.158–1.538; *p* < 0.001; q < 0.001) for SII. A two-fold increase in LMR was associated with lower mortality (adjusted HR, 0.735; 95% CI, 0.619–0.873; *p* < 0.001; q < 0.001).

Among the nutritional biomarkers, each five-unit increase in PNI was associated with a lower mortality hazard (adjusted HR, 0.819; 95% CI, 0.746–0.900; *p* < 0.001; q < 0.001). Similarly, a two-fold increase in HALP was associated with lower mortality (adjusted HR, 0.722; 95% CI, 0.624–0.834; *p* < 0.001; q < 0.001). Thus, all six prespecified biomarker associations remained statistically significant after clinical adjustment and Benjamini–Hochberg correction.

### 3.4. Incremental Prognostic Performance

The prespecified clinical model had a Harrell C-index of 0.678 and an AIC of 2070.50 (Table 3; Supplementary Tables S2 and S3). Adding each biomarker separately to this common clinical model resulted in statistically significant likelihood-ratio improvements.

The adjusted C-index increased to 0.682 with NLR (ΔC-index, +0.004), 0.683 with PLR (ΔC-index, +0.005), 0.691 with LMR (ΔC-index, +0.012), 0.688 with SII (ΔC-index, +0.010), 0.695 with PNI (ΔC-index, +0.017), and 0.693 with HALP (ΔC-index, +0.014). The corresponding likelihood-ratio test *p* values were 0.008, 0.002, <0.001, <0.001, <0.001, and <0.001, respectively.

PNI yielded the numerically largest increase in C-index, whereas the HALP-extended model had the numerically lowest AIC (2053.26), closely followed by the PNI-extended model (2055.32). The AIC values for the NLR-, PLR-, LMR-, and SII-extended models were 2065.44, 2063.21, 2060.53, and 2057.01, respectively. These findings indicate that each biomarker added prognostic information to the clinical reference model. To formally compare model discrimination, all 15 pairwise differences in C-index were evaluated using 1000 paired bootstrap resamples. None of the pairwise differences was statistically significant; all 95% bootstrap confidence intervals included zero, and all BH-adjusted q values were ≥0.455 (Supplementary Table S8). In particular, the C-index difference between the PNI- and HALP-extended models was 0.0025 (95% bootstrap CI, −0.0113 to 0.0157; *p* = 0.735; q = 0.837). Accordingly, the observed numerical differences did not provide evidence of superiority for PNI, HALP, or any other biomarker-extended model.

### 3.5. Exploratory Component-Level Analysis of HALP

In the exploratory component-level model, simultaneous addition of hemoglobin, albumin, lymphocyte count, and platelet count significantly improved model fit compared with the clinical reference model (likelihood-ratio χ^2^ = 30.31, 4 degrees of freedom, *p* < 0.001). The component model had an AIC of 2048.19 and a Harrell C-index of 0.705, compared with 2070.50 and 0.678, respectively, for the clinical model. After mutual adjustment and adjustment for the clinical covariates, higher albumin was independently associated with lower mortality (HR per 0.5-g/dL increase, 0.769; 95% CI, 0.672–0.880; *p* < 0.001; q < 0.001), whereas a two-fold increase in platelet count was associated with higher mortality (HR, 1.542; 95% CI, 1.058–2.248; *p* = 0.024; q = 0.048). Hemoglobin (HR per 1-g/dL increase, 0.982; 95% CI, 0.917–1.051; *p* = 0.595; q = 0.595) and lymphocyte count (HR per doubling, 0.905; 95% CI, 0.722–1.133; *p* = 0.384; q = 0.511) did not retain statistically significant independent associations. VIFs were low for all variables, with a maximum value of 1.52, indicating no important multicollinearity (Supplementary Table S9).

### 3.6. Time-Varying Biomarker Associations

The biomarker × log-time interaction *p* values were 0.044 for NLR, 0.053 for PLR, 0.030 for LMR, 0.044 for SII, 0.011 for PNI, and 0.029 for HALP. After Benjamini–Hochberg correction across the six interaction tests, none met the prespecified significance threshold (all adjusted q values approximately 0.053; Supplementary Table S1). The corresponding time-specific estimates were therefore interpreted as exploratory evidence of potential temporal variation rather than confirmatory evidence of non-proportional biomarker effects.

The time-specific estimates displayed a consistent descriptive pattern in which biomarker associations were strongest during early follow-up and gradually attenuated thereafter. At 3 months after diagnosis, a two-fold increase was associated with higher mortality for NLR (HR, 1.432; 95% CI, 1.158–1.769), PLR (HR, 1.630; 95% CI, 1.243–2.138), and SII (HR, 1.517; 95% CI, 1.252–1.837). Conversely, higher LMR (HR, 0.643; 95% CI, 0.524–0.788), PNI per five-unit increase (HR, 0.732; 95% CI, 0.644–0.833), and HALP per doubling (HR, 0.624; 95% CI, 0.512–0.760) were associated with lower mortality.

The associations generally remained evident at 6 months and were further attenuated by 12 months. At 12 months, the confidence interval for NLR included 1.00, whereas the corresponding intervals for PLR, LMR, SII, PNI, and HALP remained on their respective sides of the null value. By 24 months, the confidence intervals for all biomarker-specific estimates included 1.00, and the estimates were further attenuated at 36 months. At 36 months, the HRs were 1.000 for NLR, 1.030 for PLR, 0.978 for LMR, 1.077 for SII, 0.971 for PNI, and 0.909 for HALP, with all corresponding confidence intervals including 1.00. Overall, the exploratory time-specific estimates suggested that baseline systemic inflammatory burden and nutritional reserve may be more strongly associated with early mortality after diagnosis, whereas these associations appeared less pronounced at later follow-up. The number of patients remaining at risk was 118 at 12 months, 47 at 24 months, and 26 at 36 months. Therefore, the wider confidence intervals and loss of statistical significance at the later time points may partly reflect reduced precision resulting from the progressively smaller risk sets and should not be interpreted as evidence that the biological relevance of the biomarkers had disappeared.

Because the cohort included both nonmetastatic and metastatic disease, exploratory models assessed whether biomarker associations varied according to metastatic status while jointly accounting for time-dependent biomarker and metastatic-status effects. Global effect-modification tests were nominally significant for NLR (*p* = 0.023) and SII (*p* = 0.014), whereas the corresponding *p* values were 0.051 for PLR, 0.090 for LMR, 0.329 for PNI, and 0.054 for HALP. None of the six global interaction tests remained significant after Benjamini–Hochberg correction (all q ≥ 0.068). Results were similar in sensitivity models allowing all clinical covariates to vary with log-time, in which the lowest adjusted q value was 0.054. Thus, there was no multiplicity-adjusted evidence that metastatic status robustly modified the biomarker–mortality associations after temporal variation was considered, although clinically relevant disease-setting heterogeneity cannot be excluded (Supplementary Table S4).

In the complementary subgroup-specific analyses, the nonmetastatic subgroup included 138 patients and 112 deaths, whereas the metastatic subgroup included 117 patients and 115 deaths. Within the nonmetastatic subgroup, none of the six biomarkers was significantly associated with overall survival after adjustment for age, sex, and ECOG performance status; the adjusted HRs ranged from 0.943 to 1.018, and all BH-adjusted q values were 0.950. Within the metastatic subgroup, higher NLR, PLR, and SII were associated with increased mortality, whereas higher LMR, PNI, and HALP were associated with lower mortality; all six associations remained significant after multiplicity correction (all q < 0.001). Specifically, the adjusted HRs were 1.681 for NLR, 1.864 for PLR, 0.621 for LMR, 1.763 for SII, 0.753 for PNI, and 0.581 for HALP. These subgroup-specific estimates were considered exploratory and should not be interpreted as demonstrating differential effects between the two disease settings because the formal effect-modification tests did not remain significant after multiplicity correction (Supplementary Table S6).

### 3.7. Internal Validation and Sensitivity Analyses

**Figure 3:**
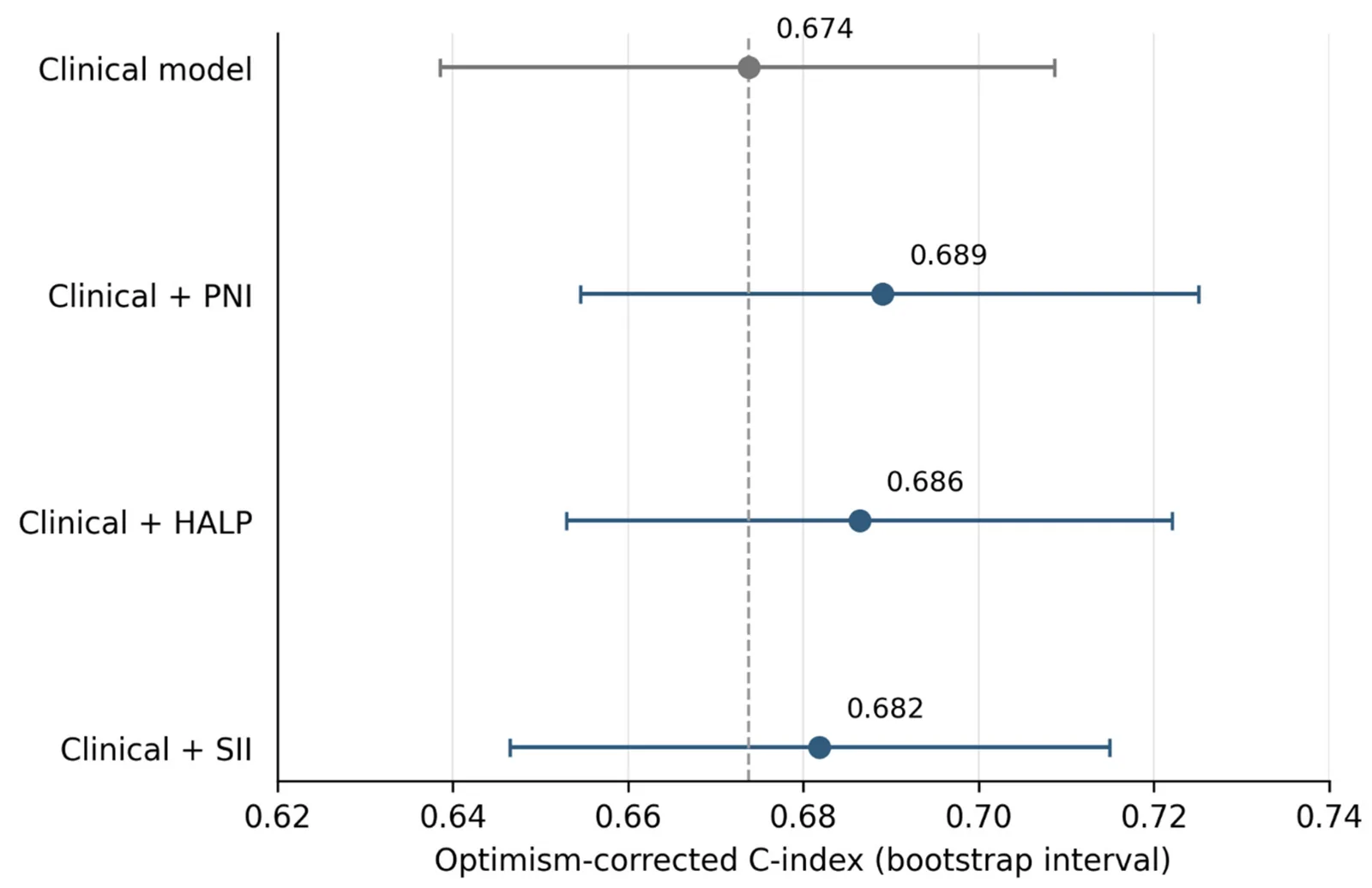
Optimism-corrected discrimination of the clinical and biomarker-extended Cox models. Points represent optimism-corrected Harrell C-index estimates, and horizontal lines represent bootstrap intervals obtained from 1000 resamples. The clinical model included age, sex, Eastern Cooperative Oncology Group performance status, and metastatic disease at diagnosis. The dashed vertical line indicates the optimism-corrected C-index of the clinical model. The PNI-extended model yielded the numerically highest optimism-corrected C-index; however, the absolute differences were modest, the bootstrap intervals substantially overlapped, and formal paired bootstrap comparisons did not identify a statistically significant difference between any of the biomarker-extended models.

In the 1000-sample bootstrap internal validation, the apparent C-index of the clinical model was 0.678, with a mean optimism of 0.005 and an optimism-corrected C-index of 0.674 (bootstrap interval, 0.639–0.709) (Supplementary Table S2; Figure 3). The optimism-corrected C-index was 0.689 (0.655–0.725) for the clinical model plus PNI, 0.686 (0.653–0.722) for the clinical model plus HALP, and 0.682 (0.647–0.715) for the clinical model plus SII. Mean optimism was low across the models, ranging from 0.005 to 0.006, and all 1000 bootstrap resamples converged successfully.

Influence-diagnostic sensitivity analyses were performed for the PNI-, HALP-, and SII-extended models (Supplementary Table S5). Thirty observations were flagged in the PNI model, 29 in the HALP model, and 26 in the SII model according to the prespecified DFBETA and generalized Cook-type thresholds. After excluding the flagged observations, the association between PNI and mortality remained significant, with an adjusted HR of 0.759 (95% CI, 0.682–0.844; *p* < 0.001) in 225 patients. The corresponding sensitivity estimates were 0.729 (95% CI, 0.621–0.855; *p* < 0.001) for HALP in 226 patients and 1.361 (95% CI, 1.161–1.597; *p* < 0.001) for SII in 229 patients. The direction and statistical significance of all three associations were preserved after exclusion of the flagged observations.

In the additional time-dependent covariate sensitivity analyses, systemic-treatment initiation dates were available for 165 patients, and verifiable primary-tumor surgery dates were available for 112 patients. After additional adjustment for systemic treatment and surgery as time-dependent covariates, the direction and statistical significance of all six biomarker associations were preserved. The adjusted HRs were 1.213 (95% CI, 1.038–1.416; *p* = 0.015; q = 0.015) for NLR, 1.350 (95% CI, 1.106–1.647; *p* = 0.003; q = 0.004) for PLR, 0.756 (95% CI, 0.635–0.901; *p* = 0.002; q = 0.003) for LMR, 1.317 (95% CI, 1.141–1.520; *p* < 0.001; q < 0.001) for SII, 0.828 (95% CI, 0.752–0.910; *p* < 0.001; q < 0.001) for PNI, and 0.728 (95% CI, 0.630–0.842; *p* < 0.001; q < 0.001) for HALP (Supplementary Table S7).

- Clinical model: 0.674 (0.639–0.709)
- Clinical + PNI: 0.689 (0.655–0.725)
- Clinical + HALP: 0.686 (0.653–0.722)
- Clinical + SII: 0.682 (0.647–0.715)

### 3.8. Kaplan–Meier Analyses

**Figure 4:**
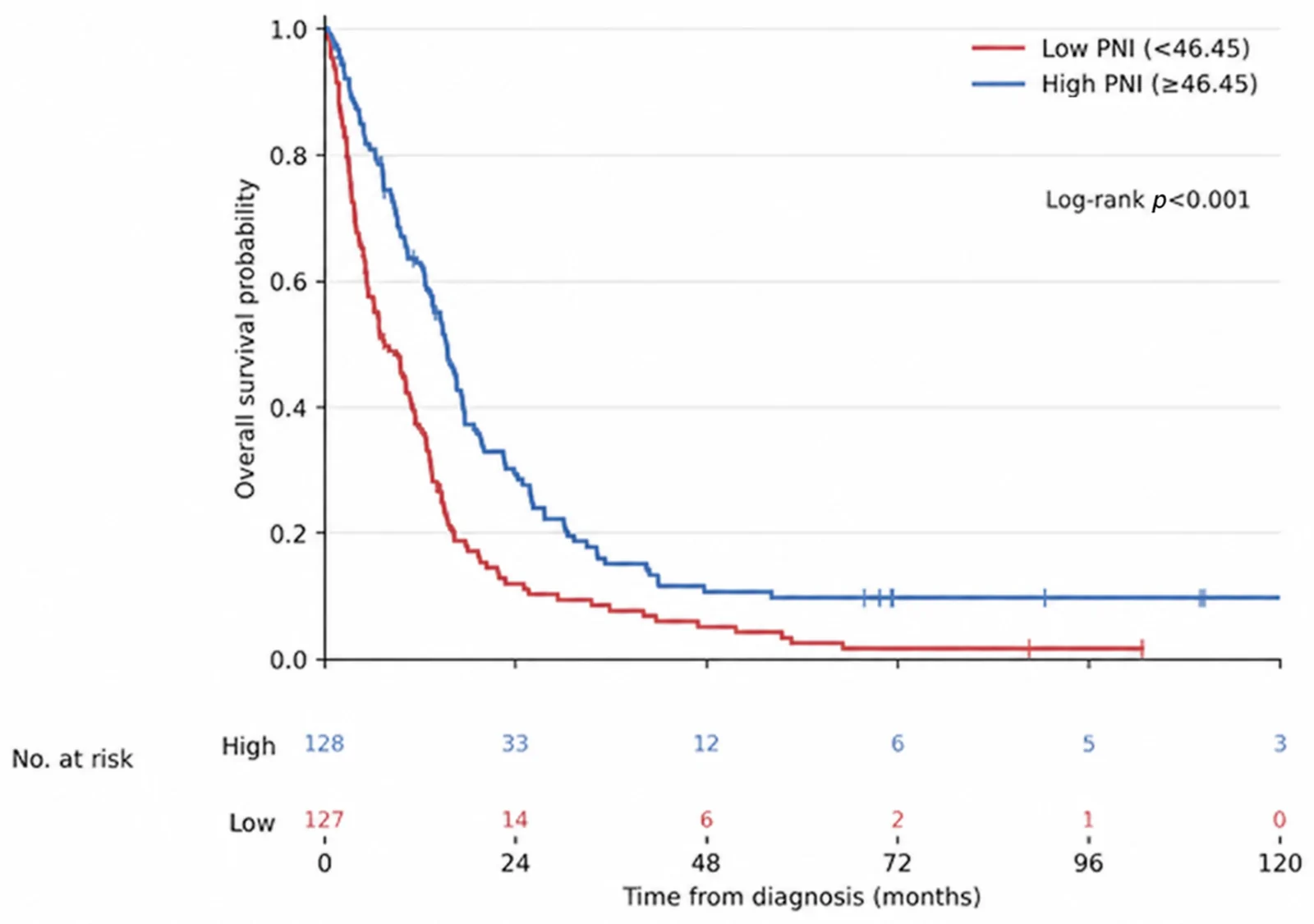
Kaplan–Meier estimates of overall survival according to the prognostic nutritional index. Patients were categorized using the cohort median PNI value of 46.45. Median overall survival was 7.62 months (95% CI, 5.36–10.71) in the low-PNI group and 15.34 months (95% CI, 12.85–17.38) in the high-PNI group. Survival distributions differed significantly between the groups (log-rank *p* < 0.001). Vertical marks indicate censored observations. The numbers below the plot show patients remaining at risk.

For descriptive Kaplan–Meier analyses, patients were divided according to the cohort median values of PNI, HALP, and SII (Figure 4, Supplementary Figures S1 and S2).

Using the median PNI value of 46.45, the low-PNI group included 127 patients, of whom 121 died. Median OS was 7.62 months (95% CI, 5.36–10.71) in the low-PNI group and 15.34 months (95% CI, 12.85–17.38) in the high-PNI group (*n* = 128; 106 deaths). The survival distributions differed significantly between the groups (log-rank *p* < 0.001).

Using the median HALP value of 22.92, the low-HALP group included 127 patients and the high-HALP group included 128 patients. Median OS was 6.93 months (95% CI, 5.29–10.32) in the low-HALP group and 15.11 months (95% CI, 12.68–16.66) in the high-HALP group; survival distributions differed significantly between the groups (log-rank *p* < 0.001).

Using the median SII value of 928.31, the low-SII group included 127 patients and the high-SII group included 128 patients. Median OS was 14.85 months (95% CI, 12.39–17.18) in the low-SII group and 8.54 months (95% CI, 5.29–11.01) in the high-SII group. Survival distributions differed significantly between the groups (log-rank *p* < 0.001), with shorter OS in the high-SII group, consistent with the continuous Cox regression findings.

Overall, the Kaplan–Meier analyses provided a consistent graphical description of the continuous-model results: lower nutritional reserve and higher systemic inflammatory burden were associated with poorer survival. These median-based analyses were used for visualization only and did not replace the prespecified continuous-variable Cox models.

## 4. Discussion

In this retrospective cohort of patients with gastric adenocarcinoma, all six inflammatory and nutritional biomarkers remained associated with overall survival when evaluated separately after adjustment for the same prespecified parsimonious clinical factors. Higher NLR, PLR, and SII were associated with increased mortality, whereas higher LMR, PNI, and HALP were associated with lower mortality, with all biomarker-specific associations remaining significant after Benjamini–Hochberg correction. Importantly, this study extended beyond conventional association testing by comparing incremental model performance, examining time-varying effects, and internally validating the leading biomarker-extended models. The findings did not establish the superiority of a single biomarker; rather, they indicate that systemic inflammatory burden and host reserve provide additional, modest prognostic information, with exploratory evidence of potential temporal variation beyond routinely available clinical factors.

The directions of the inflammatory biomarker associations were biologically plausible. Cancer-associated inflammation may facilitate angiogenesis, immune evasion, tumor-cell survival, invasion, and metastatic spread, while simultaneously contributing to systemic deterioration [5,6]. Neutrophilia may reflect both inflammatory activation and suppression of effective antitumor immunity; thrombocytosis may contribute to tumor-cell protection and metastatic dissemination; and lymphopenia may indicate impaired adaptive immune competence. Monocyte-related indices may additionally reflect activation of myeloid pathways involved in tumor progression. These mechanisms provide a coherent biological basis for the adverse associations of higher NLR, PLR, and SII and the favorable association of higher LMR observed in our cohort.

Our findings are consistent with the broader evidence supporting inflammation-based prognostic indices in solid tumors [7]. SII was initially proposed as an integrated measure of neutrophil, platelet, and lymphocyte counts [8], and a 2024 meta-analysis of 30 gastric cancer studies reported that elevated SII was associated with poorer overall survival, with a pooled hazard ratio of 1.53 [9]. In our analysis, each two-fold increase in SII was associated with a 33.5% higher mortality hazard after clinical adjustment. The numerical effect sizes should not be compared directly because the meta-analysis predominantly contrasted study-specific high and low SII categories, whereas we modeled SII continuously on the log_2_ scale. Nevertheless, the concordant direction of association supports the prognostic relevance of systemic inflammatory burden across different analytical approaches. The meta-analysis also noted substantial geographical concentration of the available evidence, emphasizing the continued need for validation in diverse real-world populations [9].

The findings for PNI and HALP extend the inflammatory framework to host nutritional, hematologic, and immune reserve. Hypoalbuminemia may reflect malnutrition, systemic inflammation, hepatic metabolic changes, and advanced disease burden, while anemia and lymphopenia may capture impaired oxygen-carrying capacity, reduced immune competence, bleeding, or treatment vulnerability [10]. PNI combines albumin and lymphocyte count [11], whereas HALP integrates hemoglobin, albumin, lymphocyte, and platelet measurements [12]. Although these indices partially overlap with inflammation-based measures, they should not be interpreted as direct measurements of nutrition alone. Rather, they represent composite markers of the patient’s systemic condition at baseline. The exploratory component-level analysis further clarified the HALP findings. When hemoglobin, albumin, lymphocyte count, and platelet count were entered simultaneously, higher albumin and lower platelet count retained independent associations with better survival, whereas hemoglobin and lymphocyte count did not remain independently significant. The low VIFs indicated that these findings were not attributable to substantial multicollinearity. These results suggest that the prognostic information captured by HALP was not distributed equally across its four components in this cohort and may have been driven predominantly by nutritional reserve and platelet-related inflammatory burden. Nevertheless, the component coefficients represent mutually adjusted associations and should not be interpreted as establishing biological causality or invalidating the potential utility of the composite HALP score.

A recent meta-analysis including 23,756 gastrectomy patients found that low PNI was associated with poorer overall survival and postoperative outcomes [13]. A contemporary early gastric cancer cohort similarly demonstrated prognostic information from both PNI and SII and reported improved model discrimination when they were combined with clinical characteristics [14]. Evidence for HALP has also expanded. Lower HALP was associated with unfavorable survival in patients undergoing curative treatment [15] and in a separate metastatic gastric cancer cohort [16]. A 2025 meta-analysis of digestive system cancers confirmed an association between HALP and survival, including within the gastric cancer subgroup [17]. Our findings are directionally consistent with this literature but broaden the clinical context by evaluating PNI and HALP within the same analytical cohort that included both nonmetastatic and metastatic disease. The recent European GASTRODATA registry analysis also linked lower PNI to worse perioperative outcomes and increased short-term mortality, supporting the relevance of host reserve beyond predominantly Asian study populations [18].

An important feature of this study was the distinction between prognostic association and incremental prognostic performance. The prespecified parsimonious clinical model had a C-index of 0.678, and the addition of individual biomarkers yielded C-indices ranging from 0.682 to 0.695. PNI produced the largest numerical increase in discrimination, whereas the HALP-extended model yielded the numerically lowest AIC, closely followed by the PNI-extended model. These findings are not contradictory: the C-index quantifies the ability of a model to rank patients according to survival risk, whereas AIC reflects relative model fit while accounting for model complexity [20]. Nevertheless, the absolute gains in discrimination were modest, and formal paired bootstrap comparisons identified no statistically significant differences among any of the biomarker-extended models. All pairwise 95% bootstrap confidence intervals included zero, including the comparison between the PNI- and HALP-extended models (ΔC-index, 0.0025; 95% CI, −0.0113 to 0.0157; *p* = 0.735; q = 0.837). Statistical significance and improved model fit do not necessarily indicate a sufficiently large improvement to support individual clinical decisions [20]. Accordingly, the formal comparisons support the conclusion that the observed numerical differences do not establish the superiority of PNI, HALP, or any other biomarker. The present findings therefore do not establish clinically meaningful improvement in individualized risk prediction or demonstrate that biomarker-informed decisions improve patient outcomes. Future studies should assess whether integrating these indices with more comprehensive clinical and tumor-related information improves prediction beyond established prognostic models. Such evaluations should include external assessment of discrimination and calibration, together with assessment of clinical utility, rather than relying on statistically significant hazard ratios alone.

The use of continuous biomarker modeling was another methodological strength. Previous prognostic biomarker studies have frequently relied on median-based, receiver-operating-characteristic-derived, or maximally selected thresholds. Although such categorization may simplify graphical presentation, it can discard information, reduce statistical efficiency, exaggerate apparent associations, and generate cohort-specific cutoffs with limited external transportability [19]. We therefore retained continuous modeling for the primary analyses and used clinically interpretable scaling: HRs per doubling for the right-skewed ratio-based biomarkers and per five-unit increase for PNI. Median-based groups were used exclusively for descriptive Kaplan–Meier visualization and were not incorporated into the primary Cox models. Consequently, the median thresholds and corresponding survival differences should not be interpreted as validated clinical cutoffs for treatment selection, surveillance, or risk classification. Clinically applicable reference ranges for these composite indices have not yet been established across heterogeneous gastric cancer populations. Thresholds reported in previous studies have varied according to disease stage, treatment setting, laboratory methods, study population, endpoint, and the statistical approach used to derive the cut-off. Consequently, proposing a reference range from the present single-center cohort would create another cohort-specific threshold rather than an externally validated clinical standard. We therefore retained the biomarkers as continuous measures and did not propose treatment or monitoring thresholds. Establishment of clinically useful reference ranges will require independent multicenter validation using standardized measurement procedures, prespecified thresholds, and assessment of whether biomarker-guided monitoring or intervention improves patient outcomes.

The exploratory time-varying analyses added an important dimension to the findings. Time-specific estimates displayed a consistent pattern of stronger biomarker associations during early follow-up and progressive attenuation thereafter; however, none of the biomarker × log-time interactions remained statistically significant after correction for multiple testing. This pattern suggests that pretreatment inflammatory burden and host reserve may predominantly capture vulnerability to early mortality, potentially reflecting advanced disease burden, aggressive tumor–host interactions, impaired physiological reserve, and limited treatment tolerance. As follow-up increases, a single baseline measurement may become less representative because subsequent treatment, disease response, progression, complications, nutritional interventions, and changes in systemic inflammatory status increasingly influence survival.

These temporal patterns should nevertheless be interpreted cautiously. Inclusion of 1.00 within the confidence intervals at later time points does not establish the absence of a prognostic association. The attenuation may reflect a genuine reduction in the relevance of the baseline biomarker, but it may also be influenced by depletion of susceptible patients, conditioning on longer survival, and the decreasing number of patients remaining at risk. In particular, only 47 and 26 patients remained at risk at 24 and 36 months, respectively; therefore, the late attenuation cannot be distinguished from reduced statistical precision and should not be interpreted as demonstrating a genuine loss of biological significance. When covariate effects vary over time, time-varying coefficient models provide a more appropriate representation than interpreting a single proportional-hazards estimate as a constant effect [21]. Accordingly, in the presence of potential nonproportionality, the conventional Cox-model HRs remain interpretable only as event-time-weighted summary associations over the observed follow-up and should not be interpreted as constant effects applicable to every patient or follow-up time point. The time-specific estimates indicate that a single baseline measurement does not carry uniform prognostic information across the entire disease course. Accordingly, the observed temporal pattern should be regarded as hypothesis-generating and requires confirmation in independent cohorts with longitudinal biomarker measurements.

The inclusion of both nonmetastatic and metastatic disease was a potential source of clinical heterogeneity. In complementary subgroup-specific models, all six biomarkers were associated with overall survival in the metastatic subgroup, whereas the corresponding estimates were close to the null and none was statistically significant in the nonmetastatic subgroup. This pattern may reflect differences in disease burden, systemic inflammation, host-reserve impairment, event distribution, treatment context, or other unmeasured characteristics between the two clinical settings. Nevertheless, differences in statistical significance between subgroup-specific estimates do not themselves establish effect modification. The formal interaction models jointly accounting for metastatic status and time-dependent associations did not identify biomarker × metastatic-status interactions that remained significant after correction for multiple testing. Nominal differences for NLR and SII attenuated after multiplicity correction, and the findings were similar when all clinical covariates were permitted to vary over time. Therefore, the stratified results should be regarded as hypothesis-generating, and limited power for interaction testing precludes either confirming or excluding clinically relevant disease-setting heterogeneity.

Treatment information provided important clinical context but was intentionally not incorporated into the primary prognostic models. Treatment allocation was nonrandom and depended on disease extent, resectability, performance status, comorbidity, molecular characteristics, calendar period, patient preference, and clinical judgment. Surgery was similarly associated with stage, operability, and treatment response. Adjustment for these variables without appropriately accounting for treatment timing and indication could introduce confounding by indication, immortal-time bias, and treatment-selection bias. Importantly, inclusion of surgery as a covariate would not compensate for incomplete tumor-stage information because surgery represents a treatment-selection variable rather than a uniform baseline measure of disease extent. These variables were therefore not incorporated into the primary prognostic models. However, in sensitivity analyses that incorporated both systemic treatment and surgery as time-dependent covariates, the direction and statistical significance of all six biomarker associations were preserved after correction for multiple testing. This finding supports the robustness of the biomarker associations to additional adjustment for the timing of major treatment exposures, but it does not eliminate residual confounding or establish causal treatment effects. Although first-line treatment status was available for the metastatic cohort, no patient received immune-checkpoint inhibitor therapy; therefore, the findings may not fully reflect outcomes under contemporary immunotherapy-containing treatment strategies. The biomarker findings should consequently be interpreted as prognostic associations in a heterogeneous real-world population rather than as evidence that these indices predict benefit from a specific treatment.

From a clinical perspective, these biomarkers have several practical advantages: they are inexpensive, widely available, and readily calculable from laboratory tests routinely obtained before treatment. Their potential value may lie in complementing—not replacing—clinical assessment by identifying patients with greater inflammatory burden or reduced host reserve who may warrant closer multidisciplinary assessment, including evaluation of nutritional and supportive-care needs. However, the present study did not evaluate whether biomarker-guided interventions improve outcomes. In the absence of externally validated reference ranges, the values should be interpreted in conjunction with the underlying laboratory measurements, disease setting, and overall clinical assessment rather than as fixed clinical thresholds. The associations observed in this study are prognostic and do not establish that these indices predict benefit from a particular treatment. In the absence of clinically validated, standardized thresholds for the relevant gastric cancer population and treatment setting, PNI, HALP, SII, and the other evaluated indices should not be used in isolation to select, withhold, or modify anticancer treatment.

Several aspects strengthen the analysis. All biomarker models were fitted in the same complete-case cohort and compared with an identical prespecified clinical reference model, reducing variation attributable to changing analytical samples or adjustment sets. Biomarkers were primarily retained as continuous variables, multiple testing was addressed using Benjamini–Hochberg correction, and model performance was assessed using complementary measures rather than *p* values alone. Proportional-hazards assumptions were formally evaluated, and exploratory time-specific estimates were reported when nominal evidence of potential temporal variation was observed.

Bootstrap validation showed low optimism and complete convergence across all 1000 resamples. Furthermore, influence-diagnostic sensitivity analyses preserved the direction and statistical significance of the leading biomarker associations after removal of observations exerting comparatively greater influence.

Overall, the findings support a multidimensional interpretation of circulating biomarkers in gastric adenocarcinoma. Inflammation-based indices predominantly reflect systemic inflammatory burden, whereas PNI and HALP capture broader aspects of host nutritional, hematologic, and immune reserve. These measures remained associated with survival when evaluated separately after clinical adjustment, but their incremental contribution to discrimination was modest, while exploratory time-specific estimates suggested stronger associations with mortality during early follow-up. Future studies should validate these findings in independent multicenter cohorts, incorporate standardized and longitudinal biomarker measurements, evaluate stage- and treatment-specific effects, and determine whether dynamic biomarker assessment provides clinically meaningful improvement over conventional prognostic evaluation.

### Limitations

The retrospective single-center design limits the generalizability of this study and introduces the possibility of selection bias, information bias, and residual confounding. Validation in independent multicenter cohorts is required before these findings can support routine clinical implementation; bootstrap internal validation does not establish external validity. Biomarkers were measured at a single pretreatment time point, precluding assessment of within-patient changes during treatment or follow-up. The exploratory time-varying coefficient analyses examined changes in the prognostic associations of baseline values, rather than changes in biomarker levels themselves; serial measurements are therefore needed to evaluate the additional prognostic value of biomarker trajectories. Although the biomarkers were associated with overall survival, their incremental contribution to discrimination was modest, and clinically meaningful improvement in individual risk prediction or patient outcomes from biomarker-informed decisions was not established. Future studies should evaluate their integration with more comprehensive clinical and tumor-related information, with external assessment of discrimination, calibration, and clinical utility. The inclusion of metastatic and nonmetastatic disease and different tumor locations introduced clinical and therapeutic heterogeneity. Complete stage information was unavailable for 32 nonmetastatic patients (12.5% of the total cohort), and tumor-location data were also incomplete. Consequently, adjustment for metastatic status and exploratory subgroup analyses cannot eliminate residual confounding or establish performance within specific stage- or tumor-location-defined populations. Clinically validated, standardized thresholds for these indices have not been established for the population and treatment settings represented here; the indices should not be used in isolation to select, withhold, or modify anticancer treatment. Finally, no patient received immune checkpoint inhibitor therapy. The observed associations and model performance cannot be assumed to apply to contemporary immunotherapy-containing regimens, and the study provides no evidence that these indices predict response to or benefit from immune checkpoint inhibitors.

Additional methodological limitations warrant consideration. The complete-case restriction may have introduced selection bias because the missing-data mechanism could not be formally assessed and the excluded records lacked sufficient analyzable baseline information for comparison with the final cohort. Baseline laboratory values may have been influenced by infection, bleeding, dehydration, comorbidity, medication exposure, or other nonmalignant conditions that could not be comprehensively identified. Although measurements were restricted to the prespecified seven-day window, their exact timing relative to treatment initiation or initial oncological assessment varied. Each biomarker was evaluated separately against the same clinical reference model; these analyses therefore do not establish contributions independent of the other overlapping indices. The mutually adjusted HALP component analysis remains exploratory and requires external validation. Potential nonproportionality also means that conventional Cox-model hazard ratios provide overall summary associations rather than constant effects throughout follow-up. The decreasing numbers at risk limit the precision of later time-specific estimates, while the sample size may have limited the ability to detect small differences in discrimination between models. Additional adjustment for systemic treatment and surgery as time-dependent covariates does not eliminate residual confounding or establish causal treatment effects.

## 5. Conclusions

In patients with gastric adenocarcinoma, routinely available inflammatory and nutritional biomarkers were associated with overall survival when evaluated separately after adjustment for a prespecified parsimonious set of clinical factors. Higher NLR, PLR, and SII were associated with increased mortality, whereas higher LMR, PNI, and HALP were associated with more favorable outcomes. PNI yielded the largest numerical increase in discrimination, while the HALP-extended model had the numerically lowest AIC; however, the absolute incremental gains were modest, and formal paired bootstrap comparisons did not establish the superiority of any single biomarker. Exploratory time-specific estimates suggested that biomarker associations with mortality may be stronger during early follow-up and attenuate thereafter, but this temporal pattern requires external confirmation. These indices may complement conventional clinical assessment but should not be used as stand-alone tools for treatment selection. External validation and longitudinal evaluation are required before their incorporation into routine prognostic algorithms.

## Figures and Tables

**Table 1:** Baseline clinical, hematological, and biomarker characteristics of the complete-case cohort.

Characteristic	Overall Cohort (*n* = 255)
**Age, years**	63.5 (57.2–71.1)
**Sex,** ***n* (%)**	
Female	84 (32.9)
Male	171 (67.1)
**ECOG performance status,** ***n* (%)**	
0–1	189 (74.1)
≥2	66 (25.9)
**Metastatic disease at diagnosis,** ***n* (%)**	
No	138 (54.1)
Yes	117 (45.9)
**Tumor location, *n* (%)** *	
Stomach	135 (71.8)
Gastroesophageal junction	53 (28.2)
**Primary tumor surgery,** ***n* (%) †**	
No	141 (55.7)
Yes	112 (44.3)
**Baseline hematological and nutritional parameters**	
Hemoglobin, g/dL	12.5 (10.5–14.0)
Albumin, g/dL	3.80 (3.40–4.13)
Neutrophils, ×10^9^/L	4.95 (3.92–6.80)
Lymphocytes, ×10^9^/L	1.64 (1.19–2.13)
Monocytes, ×10^9^/L	0.51 (0.41–0.71)
Platelets, ×10^9^/L	299.0 (233.5–363.5)
**Inflammatory and nutritional indices**	
NLR	3.08 (2.14–4.96)
PLR	185.5 (128.4–256.6)
LMR	3.15 (2.04–4.25)
SII	928.3 (540.9–1534.9)
PNI	46.45 (40.67–51.57)
HALP	22.92 (14.93–40.54)

**Notes:** Values are presented as median (interquartile range) unless otherwise indicated. Data were complete for the outcome, prespecified clinical model covariates, and all biomarker components in the analytical cohort. * Tumor-location data were available for 188 patients; percentages were calculated using the available-data denominator. † Primary-tumor surgery data were available for 253 patients; percentages were calculated using the available-data denominator. **Abbreviations:** ECOG, Eastern Cooperative Oncology Group; HALP, hemoglobin–albumin–lymphocyte–platelet score; LMR, lymphocyte-to-monocyte ratio; NLR, neutrophil-to-lymphocyte ratio; PLR, platelet-to-lymphocyte ratio; PNI, prognostic nutritional index; SII, systemic immune-inflammation index.

**Table 2:** Univariable Cox regression analysis of factors associated with overall survival.

Variable	Comparison/Increment	HR (95% CI)	*p* Value
Age	Per 10-year increase	1.279 (1.116–1.466)	<0.001
Sex	Male vs. female	0.969 (0.735–1.278)	0.826
ECOG performance status	≥2 vs. 0–1	2.161 (1.610–2.902)	<0.001
Metastatic disease at diagnosis	Yes vs. no	2.231 (1.704–2.919)	<0.001
NLR	Per doubling	1.358 (1.183–1.559)	<0.001
PLR	Per doubling	1.557 (1.283–1.891)	<0.001
LMR	Per doubling	0.658 (0.559–0.775)	<0.001
SII	Per doubling	1.458 (1.276–1.666)	<0.001
PNI	Per 5-unit increase	0.779 (0.714–0.850)	<0.001
HALP	Per doubling	0.665 (0.579–0.763)	<0.001

**Notes:** Univariable Cox proportional hazards regression analyses were performed in the common complete-case cohort of 255 patients, including 227 deaths. NLR, PLR, LMR, SII, and HALP were log_2_-transformed because of their right-skewed distributions; corresponding hazard ratios therefore represent a two-fold increase in the respective biomarker. PNI was modeled on its original continuous scale, and its hazard ratio represents a five-unit increase. No data-driven categorization was used in the Cox analyses. **Abbreviations:** CI, confidence interval; ECOG, Eastern Cooperative Oncology Group; HALP, hemoglobin–albumin–lymphocyte–platelet score; HR, hazard ratio; LMR, lymphocyte-to-monocyte ratio; NLR, neutrophil-to-lymphocyte ratio; PLR, platelet-to-lymphocyte ratio; PNI, prognostic nutritional index; SII, systemic immune-inflammation index.

**Table 3:** Adjusted associations and incremental prognostic performance of inflammatory and nutritional biomarkers.

Model	Adjusted HR (95% CI)	*p* Value	BH-Adjusted q Value	C-Index	ΔC-Index	AIC	LR *p* Value
Clinical model	—	—	—	0.678	Reference	2070.50	—
Clinical model + NLR	1.233 (1.059–1.435)	0.007	0.007	0.682	+0.004	2065.44	0.008
Clinical model + PLR	1.365 (1.118–1.666)	0.002	0.003	0.683	+0.005	2063.21	0.002
Clinical model + LMR	0.735 (0.619–0.873)	<0.001	<0.001	0.691	+0.012	2060.53	<0.001
Clinical model + SII	1.335 (1.158–1.538)	<0.001	<0.001	0.688	+0.010	2057.01	<0.001
Clinical model + PNI	0.819 (0.746–0.900)	<0.001	<0.001	0.695	+0.017	2055.32	<0.001
Clinical model + HALP	0.722 (0.624–0.834)	<0.001	<0.001	0.693	+0.014	2053.26	<0.001

**Notes:** Each biomarker was added separately to the same prespecified clinical model comprising age, sex, Eastern Cooperative Oncology Group performance status, and metastatic disease at diagnosis. All models were fitted in the common complete-case cohort of 255 patients with 227 deaths. NLR, PLR, LMR, SII, and HALP were log_2_-transformed; their hazard ratios represent a two-fold increase. The hazard ratio for PNI represents a five-unit increase. ΔC-index denotes the absolute change relative to the clinical model. Likelihood-ratio tests compared each biomarker-extended model with the clinical model. Benjamini–Hochberg correction was applied across the six biomarker-specific comparisons. For biomarkers with nominal evidence of potentially non-proportional effects, the conventional Cox-model HR represents an overall summary association across follow-up and should not be interpreted as necessarily time-invariant. Exploratory time-specific estimates are presented in Supplementary Table S1. **Abbreviations:** AIC, Akaike information criterion; BH, Benjamini–Hochberg; CI, confidence interval; ECOG, Eastern Cooperative Oncology Group; HALP, hemoglobin–albumin–lymphocyte–platelet score; HR, hazard ratio; LMR, lymphocyte-to-monocyte ratio; LR, likelihood ratio; NLR, neutrophil-to-lymphocyte ratio; PLR, platelet-to-lymphocyte ratio; PNI, prognostic nutritional index; SII, systemic immune-inflammation index.

## Data Availability

The data presented in this study are available from the corresponding author upon reasonable request. The data are not publicly available because they contain patient-level clinical information subject to institutional, ethical, and privacy restrictions.

## Supplementary Materials

The following supporting information can be downloaded at: https://www.mdpi.com/article/10.3390/diagnostics16213459/s1. Table S1: Exploratory time-varying adjusted associations of inflammatory and nutritional biomarkers with overall survival; Table S2: Bootstrap internal validation of model discrimination; Table S3: Multivariable Cox regression results for the prespecified clinical model; Table S4: Exploratory assessment of effect modification by metastatic status after accounting for time-varying associations; Table S5: Influence-diagnostic sensitivity analyses for the leading biomarker models; Table S6: Exploratory subgroup-specific adjusted associations of inflammatory and nutritional biomarkers with overall survival according to metastatic status at diagnosis; Table S7: Sensitivity analyses additionally adjusted for systemic treatment and surgery as time-dependent covariates; Table S8: Paired bootstrap comparisons of Harrell C-indices among biomarker-extended models; Table S9: Mutually adjusted associations of the individual HALP components with overall survival; Figure S1: Kaplan–Meier estimates of overall survival according to the hemoglobin–albumin–lymphocyte–platelet score. Patients were categorized using the cohort median HALP score of 22.92. Median overall survival was 6.93 months (95% CI, 5.29–10.32) in the low-HALP group and 15.11 months (95% CI, 12.68–16.66) in the high-HALP group. Overall survival differed significantly between the groups (log-rank p<0.001). Vertical marks indicate censored observations, and the numbers below the plot show patients re-maining at risk; Figure S2: Kaplan–Meier estimates of overall survival according to the systemic immune-inflammation index. Patients were categorized using the cohort median SII value of 928.31. Median overall sur-vival was 14.85 months (95% CI, 12.39–17.18) in the low-SII group and 8.54 months (95% CI, 5.29–11.01) in the high-SII group. Overall survival differed significantly between the groups (log-rank p<0.001). Vertical marks indicate censored observations, and the numbers below the plot show patients remaining at risk.
