# Supplementary material for: Inflammatory and Nutritional Biomarkers for Overall Survival in Gastric Adenocarcinoma: Comparative Performance and Exploratory Temporal Patterns
Source: Diagnostics (Basel). 2026 Oct 23;16(21):3459. doi: 10.3390/diagnostics16213459 (PMC13762303; doi:10.3390/diagnostics16213459)
Supplement: Supplementary file 1 [file diagnostics-16-03459-s001.zip › diagnostics-4564237-supplementary.pdf]

Supplementary Materials

Inflammatory and Nutritional Biomarkers for Overall Survival in Gastric Adenocarcinoma: Comparative Performance and Exploratory Temporal Patterns

Erdoğan Şeyran, Azer Gökmen, Çağlar Köseoğlu and Emre Hafızoğlu

Table S1. Exploratory time-varying adjusted associations of inflammatory and nutritional biomarkers with overall survival.

Biomarker	Biomarker × log-time p value	BH-adjusted q value	3-month HR (95% CI)	6-month HR (95% CI)	12-month HR (95% CI)	24-month HR (95% CI)	36-month HR (95% CI)
NLR, per doubling	0.044	0.053	1.432 (1.158–1.769)	1.295 (1.101–1.523)	1.172 (0.993–1.382)	1.060 (0.852–1.319)	1.000 (0.769–1.300)
PLR, per doubling	0.053	0.053	1.630 (1.243–2.138)	1.434 (1.165–1.766)	1.262 (1.016–1.567)	1.110 (0.830–1.486)	1.030 (0.726–1.463)
LMR, per doubling	0.030	0.053	0.643 (0.524–0.788)	0.722 (0.610–0.856)	0.812 (0.669–0.986)	0.913 (0.703–1.187)	0.978 (0.716–1.335)
SII, per doubling	0.044	0.053	1.517 (1.252–1.837)	1.379 (1.188–1.600)	1.253 (1.068–1.470)	1.139 (0.918–1.414)	1.077 (0.831–1.397)
PNI, per 5-unit increase	0.011	0.053	0.732 (0.644–0.833)	0.792 (0.718–0.874)	0.857 (0.776–0.947)	0.928 (0.812–1.060)	0.971 (0.827–1.141)
HALP, per doubling	0.029	0.053	0.624 (0.512–0.760)	0.693 (0.596–0.806)	0.770 (0.657–0.901)	0.855 (0.691–1.058)	0.909 (0.704–1.175)

Notes: Time-specific hazard ratios were estimated using extended Cox models incorporating biomarker × log-time interaction terms. Each model was adjusted for age, sex, Eastern Cooperative Oncology Group performance status, and metastatic disease at diagnosis. Hazard ratios for NLR, PLR, LMR, SII, and HALP represent a two-fold increase in the corresponding biomarker; hazard ratios for PNI represent a five-unit increase. Benjamini–Hochberg correction was applied across the six biomarker × log-time interaction tests. None of the interactions met the prespecified significance threshold after correction for multiple testing. The time-specific estimates should therefore be interpreted as exploratory descriptions of potential temporal patterns rather than confirmatory evidence of time-varying effects. **Abbreviations:** BH, Benjamini–Hochberg; CI, confidence interval; HALP, hemoglobin–albumin–lymphocyte–platelet score; HR, hazard ratio; LMR, lymphocyte-to-monocyte ratio; NLR, neutrophil-to-lymphocyte ratio; PLR, platelet-to-lymphocyte ratio; PNI, prognostic nutritional index; SII, systemic immune-inflammation index.

Table S2. Bootstrap internal validation of model discrimination.

Model	Apparent C-index	Mean optimism	Optimism-corrected C-index	Bootstrap interval
Clinical model	0.678	0.005	0.674	0.639–0.709
Clinical model + PNI	**0.695**	0.006	**0.689**	0.655–0.725
Clinical model + HALP	0.693	0.006	0.686	0.653–0.722
Clinical model + SII	0.688	0.006	0.682	0.647–0.715

Notes: Internal validation was performed using 1,000 bootstrap resamples. In each bootstrap resample, the model was refitted, and its discrimination was evaluated both in the bootstrap sample and in the original cohort. Optimism was calculated as the mean difference between bootstrap-sample and original-cohort performance. The optimism-corrected C-index was obtained by subtracting mean optimism from the apparent C-index. Bootstrap intervals represent the 2.5th and 97.5th percentiles of the optimism-corrected estimates. All 1,000 bootstrap resamples converged successfully. Values were rounded independently; therefore, subtraction using the displayed values may not exactly reproduce the reported optimism-corrected C-index. The clinical model included age, sex, Eastern Cooperative Oncology Group performance status, and metastatic disease at diagnosis. PNI was modeled per five-unit increase; HALP and SII were log_2-transformed. **Abbreviations:** HALP, hemoglobin–albumin–lymphocyte–platelet score; PNI, prognostic nutritional index; SII, systemic immune-inflammation index.

Table S3. Multivariable Cox regression results for the prespecified clinical model.

Variable	Comparison/increment	Adjusted HR (95% CI)	p value
Age	Per 10-year increase	1.255 (1.094–1.439)	0.001
Sex	Male vs. female	1.171 (0.879–1.561)	0.282
ECOG performance status	≥2 vs. 0–1	1.943 (1.424–2.651)	<0.001
Metastatic disease at diagnosis	Yes vs. no	2.314 (1.765–3.035)	<0.001

Model performance

Metric	Value
Patients included	255
Death events	227
Harrell's C-index	0.678
Akaike information criterion	2070.50

Notes: The clinical model was prespecified and included age, sex, Eastern Cooperative Oncology Group performance status, and metastatic disease at diagnosis. Age was modeled continuously and scaled per 10-year increase. This model served as the reference model for all biomarker-specific comparisons. Each inflammatory or nutritional biomarker was added separately to this same clinical model. **Abbreviations:** AIC, Akaike information criterion; CI, confidence interval; ECOG, Eastern Cooperative Oncology Group; HR, hazard ratio.

Table S4. Exploratory assessment of effect modification by metastatic status after accounting for time-varying associations.

Biomarker	Targeted temporal model LR χ^2 (2 df)	p value	BH-adjusted q value	Full temporal sensitivity model LR χ^2 (2 df)*	Sensitivity p value*	Sensitivity q value*
NLR, per doubling	7.573	0.023	0.068	8.035	0.018	0.054
PLR, per doubling	5.958	0.051	0.081	5.382	0.068	0.090
LMR, per doubling	4.826	0.090	0.107	6.139	0.046	0.090
SII, per doubling	8.529	0.014	0.068	8.572	0.014	0.054
PNI, per 5-unit increase	2.224	0.329	0.329	2.021	0.364	0.364
HALP, per doubling	5.839	0.054	0.081	5.191	0.075	0.090

Notes: Global effect modification was evaluated using a two-degree-of-freedom likelihood-ratio test jointly assessing the static biomarker × metastatic-status interaction and the biomarker × metastatic-status × log-time interaction. The targeted temporal models were adjusted for age, sex, Eastern Cooperative Oncology Group performance status, metastatic disease at diagnosis, and the corresponding biomarker and included biomarker × log-time and metastatic-status × log-time terms. Benjamini–Hochberg correction was applied across the six global effect-modification tests. *In the full temporal sensitivity models, age, sex, and Eastern Cooperative Oncology Group performance status were additionally permitted to vary with log-time. All targeted and sensitivity models converged successfully. No global effect-modification test remained statistically significant after correction for multiple testing. **Abbreviations:** BH, Benjamini–Hochberg; ECOG, Eastern Cooperative Oncology Group; HALP, hemoglobin–albumin–lymphocyte–platelet score; LMR, lymphocyte-to-monocyte ratio; LR, likelihood ratio; NLR, neutrophil-to-lymphocyte ratio; PLR, platelet-to-lymphocyte ratio; PNI, prognostic nutritional index; SII, systemic immune-inflammation index.

Table S5. Influence-diagnostic sensitivity analyses for the leading biomarker models.

Model	Full-cohort adjusted HR (95% CI)	Observations flagged, n	Sensitivity cohort, n	Adjusted HR after exclusion (95% CI)	p value
PNI, per 5-unit increase	0.819 (0.746–0.900)	30	225	0.759 (0.682–0.844)	<0.001
HALP, per doubling	0.722 (0.624–0.834)	29	226	0.729 (0.621–0.855)	<0.001
SII, per doubling	1.335 (1.158–1.538)	26	229	1.361 (1.161–1.597)	<0.001

Notes: Influence diagnostics were evaluated separately for each biomarker-specific model using DFBETAs and a generalized Cook-type measure. Observations were flagged when the maximum absolute DFBETA exceeded **$2/\sqrt{n}$ = 0.125** or when the generalized Cook-type measure exceeded **4/n = 0.0157**. Sensitivity models were refitted after excluding observations flagged within the corresponding model. All models were adjusted for age, sex, Eastern Cooperative Oncology Group performance status, and metastatic disease at diagnosis. PNI was modeled per five-unit increase. HALP and SII were $\log_2$-transformed; their hazard ratios

represent a two-fold increase. Flagged observations were not considered erroneous records but observations exerting comparatively greater influence on model estimates. **Abbreviations:** CI, confidence interval; DFBETA, standardized change in a regression coefficient following observation deletion; HALP, hemoglobin–albumin–lymphocyte–platelet score; HR, hazard ratio; PNI, prognostic nutritional index; SII, systemic immune-inflammation index.

Table S6. Exploratory subgroup-specific adjusted associations of inflammatory and nutritional biomarkers with overall survival according to metastatic status at diagnosis.

Biomarker	Nonmetastatic subgroup adjusted HR (95% CI)	p value	BH-adjusted q value	Metastatic subgroup adjusted HR (95% CI)	p value	BH-adjusted q value
NLR, per doubling	0.944 (0.758–1.175)	0.604	0.950	1.681 (1.326–2.130)	<0.001	<0.001
PLR, per doubling	0.960 (0.720–1.280)	0.782	0.950	1.864 (1.396–2.488)	<0.001	<0.001
LMR, per doubling	0.992 (0.762–1.291)	0.950	0.950	0.621 (0.492–0.783)	<0.001	<0.001
SII, per doubling	1.018 (0.830–1.248)	0.864	0.950	1.763 (1.417–2.194)	<0.001	<0.001
PNI, per 5-unit increase	0.952 (0.824–1.099)	0.500	0.950	0.753 (0.664–0.854)	<0.001	<0.001
HALP, per doubling	0.943 (0.759–1.171)	0.592	0.950	0.581 (0.473–0.714)	<0.001	<0.001

Notes: Separate Cox proportional hazards models were fitted within the nonmetastatic subgroup (n=138; 112 deaths) and the metastatic subgroup (n=117; 115 deaths). Each model was adjusted for age (per 10-year increase), sex, and Eastern Cooperative Oncology Group performance status and included one biomarker at a time. Metastatic status was omitted because it was constant within each subgroup. NLR, PLR, LMR, SII, and HALP were modeled on the log2 scale, so their hazard ratios represent a twofold increase; the PNI hazard ratio represents a 5-unit increase. Benjamini–Hochberg correction was applied separately across the six biomarker tests within each subgroup. These analyses were exploratory. Differences in statistical significance between the subgroup-specific estimates should not be interpreted as evidence of effect modification; formal between-group heterogeneity was evaluated using the interaction analyses reported in Table S4. **Abbreviations:** BH, Benjamini–Hochberg; CI, confidence interval; ECOG, Eastern Cooperative Oncology Group; HALP, hemoglobin–albumin–lymphocyte–platelet score; HR, hazard ratio; LMR, lymphocyte-to-monocyte ratio; NLR, neutrophil-to-lymphocyte ratio; PLR, platelet-to-lymphocyte ratio; PNI, prognostic nutritional index; SII, systemic immune-inflammation index.

Table S7. Sensitivity analyses additionally adjusted for systemic treatment and surgery as time-dependent covariates.

Biomarker	Scale	Adjusted HR (95% CI)	p value	BH-adjusted q value
NLR	Per doubling	1.213 (1.038–1.416)	0.015	0.015
PLR	Per doubling	1.350 (1.106–1.647)	0.003	0.004
LMR	Per doubling	0.756 (0.635–0.901)	0.002	0.003
SII	Per doubling	1.317 (1.141–1.520)	<0.001	<0.001
PNI	Per 5-unit increase	0.828 (0.752–0.910)	<0.001	<0.001
HALP	Per doubling	0.728 (0.630–0.842)	<0.001	<0.001

Notes: Each biomarker was evaluated in a separate extended Cox model adjusted for age (per 10-year increase), sex, Eastern Cooperative Oncology Group performance status, metastatic disease at diagnosis, receipt of systemic treatment as a time-dependent covariate, and surgery as a time-dependent covariate. Systemic treatment status changed from 0 to 1 at the earliest recorded initiation date of neoadjuvant, adjuvant, or first-line palliative systemic therapy; surgery status changed from 0 to 1 on the recorded surgery date. Among the 255 patients, verifiable dates for time-dependent coding were available for systemic-treatment initiation in 165 patients and primary-tumor surgery in 112 patients. NLR, PLR, LMR, SII, and HALP were modeled on the log2 scale, so their hazard ratios represent a twofold increase; the PNI hazard ratio represents a 5-unit increase. Benjamini–Hochberg correction was applied across the six biomarker tests. Treatment and surgery coefficients are not presented because these variables were included solely as time-dependent adjustment covariates and were not evaluated as causal treatment effects. **Abbreviations:** BH, Benjamini–Hochberg; CI, confidence interval; ECOG, Eastern Cooperative Oncology Group; HALP, hemoglobin–albumin–lymphocyte–platelet score; HR, hazard ratio; LMR, lymphocyte-to-monocyte ratio; NLR, neutrophil-to-lymphocyte ratio; PLR, platelet-to-lymphocyte ratio; PNI, prognostic nutritional index; SII, systemic immune-inflammation index.

Table S8. Paired bootstrap comparisons of Harrell C-indices among biomarker-extended models.

Model comparison	C-index of Model 1	C-index of Model 2	ΔC-index (Model 1 − Model 2)	95% bootstrap CI	p value	BH-adjusted q value
NLR vs. PLR	0.682	0.683	−0.0009	−0.0140–0.0087	0.745	0.837
NLR vs. LMR	0.682	0.691	−0.0087	−0.0209–0.0033	0.182	0.455
NLR vs. SII	0.682	0.688	−0.0062	−0.0188–0.0019	0.134	0.455
NLR vs. PNI	0.682	0.695	−0.0132	−0.0302–0.0006	0.066	0.455
NLR vs. HALP	0.682	0.693	−0.0107	−0.0277–0.0026	0.146	0.455
PLR vs. LMR	0.683	0.691	−0.0078	−0.0209–0.0086	0.370	0.693
PLR vs. SII	0.683	0.688	−0.0053	−0.0166–0.0035	0.264	0.565
PLR vs. PNI	0.683	0.695	−0.0123	−0.0272–0.0030	0.152	0.455
PLR vs. HALP	0.683	0.693	−0.0098	−0.0197–0.0001	0.058	0.455
LMR vs. SII	0.691	0.688	+0.0025	−0.0140–0.0146	0.917	0.917
LMR vs. PNI	0.691	0.695	−0.0045	−0.0222–0.0105	0.541	0.755
LMR vs. HALP	0.691	0.693	−0.0020	−0.0203–0.0128	0.781	0.837
SII vs. PNI	0.688	0.695	−0.0070	−0.0224–0.0106	0.492	0.755
SII vs. HALP	0.688	0.693	−0.0045	−0.0158–0.0073	0.553	0.755
PNI vs. HALP	0.695	0.693	+0.0025	−0.0113–0.0157	0.735	0.837

Notes: Each biomarker-extended model included age, sex, Eastern Cooperative Oncology Group performance status, metastatic disease at diagnosis, and one biomarker. Direct differences in Harrell C-indices were evaluated using 1,000 paired bootstrap resamples. Within each bootstrap resample, all six biomarker-extended models were refitted using the same resampled patients, and the C-index difference was calculated for each model pair. The 95% confidence intervals were derived from the percentile distribution of the paired bootstrap differences. Two-sided p values evaluated whether the paired C-index difference included zero. Benjamini–Hochberg correction was applied across the 15 pairwise comparisons. All 1,000 bootstrap resamples converged successfully. None of the pairwise C-index differences was statistically significant before or after correction for multiple testing. **Abbreviations:** BH, Benjamini–Hochberg; CI, confidence interval; ECOG, Eastern Cooperative Oncology Group; HALP, hemoglobin–albumin–lymphocyte–platelet score; LMR, lymphocyte-to-monocyte ratio; NLR, neutrophil-to-lymphocyte ratio; PLR, platelet-to-lymphocyte ratio; PNI, prognostic nutritional index; SII, systemic immune-inflammation index.

Table S9. Mutually adjusted associations of the individual HALP components with overall survival.

HALP component	Unit of increase	Adjusted HR	95% CI	p value	BH-adjusted q value
Hemoglobin	1 g/dL	0.982	0.917–1.051	0.595	0.595
Albumin	0.5 g/dL	0.769	0.672–0.880	<0.001	<0.001
Lymphocyte count	Two-fold increase	0.905	0.722–1.133	0.384	0.511
Platelet count	Two-fold increase	1.542	1.058–2.248	0.024	0.048

Notes: All four HALP components were entered simultaneously into the same Cox proportional hazards model, which was additionally adjusted for age, sex, ECOG performance status, and metastatic disease at diagnosis. Lymphocyte and platelet counts were $\log_2$-transformed. The component model had an AIC of 2048.19 and a Harrell C-index of 0.705. Compared with the clinical reference model, the likelihood-ratio χ^2 was 30.31 with 4 degrees of freedom ($p<0.001$). The maximum variance inflation factor was 1.52. Benjamini–Hochberg correction was applied across the four component-specific tests. **Abbreviations:** AIC, Akaike information criterion; BH, Benjamini–Hochberg; CI, confidence interval; C-index, Harrell's concordance index; ECOG, Eastern Cooperative Oncology Group; HALP, hemoglobin–albumin–lymphocyte–platelet score; HR, hazard ratio; VIF, variance inflation factor.

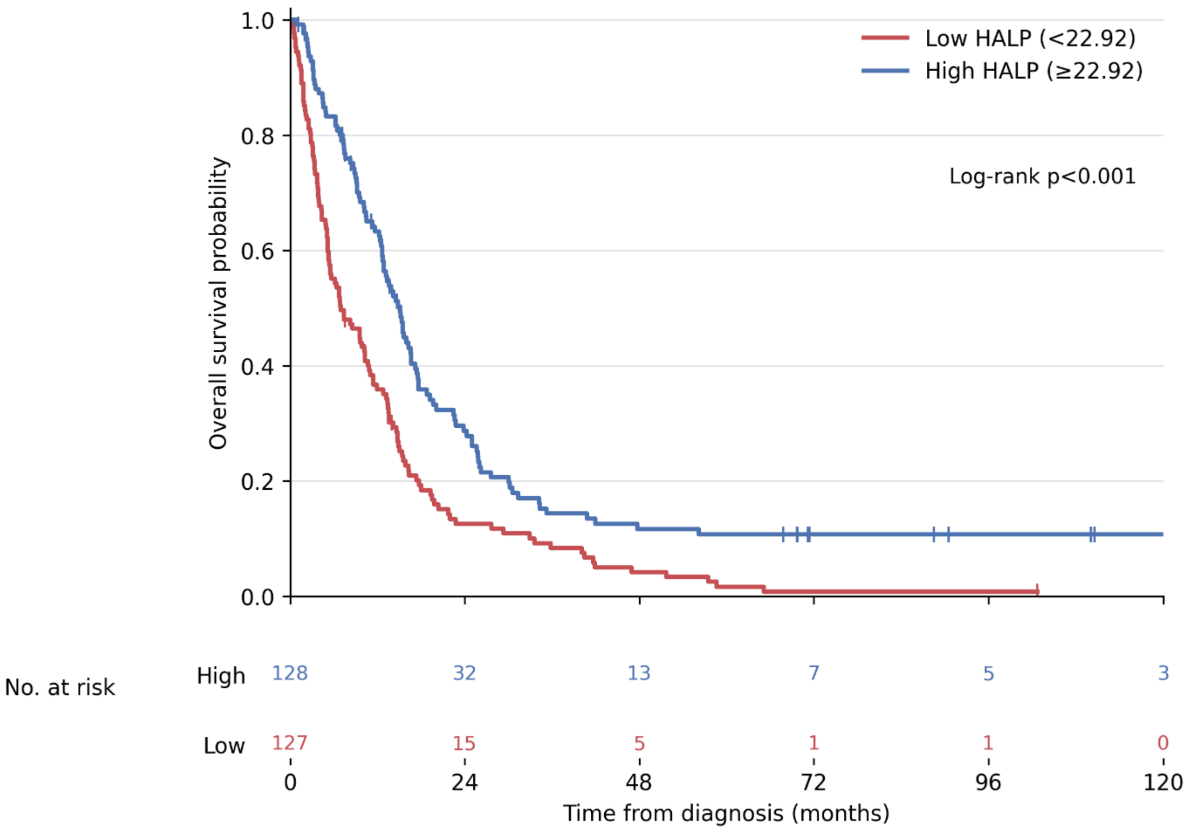

Figure S1. Kaplan–Meier estimates of overall survival according to the hemoglobin–albumin–lymphocyte–platelet score. Patients were categorized using the cohort median HALP score of 22.92. Median overall survival was 6.93 months (95% CI, 5.29–10.32) in the low-HALP group and 15.11 months (95% CI, 12.68–16.66) in the high-HALP group. Overall survival differed significantly between the groups (log-rank p<0.001). Vertical marks indicate censored observations, and the numbers below the plot show patients remaining at risk.

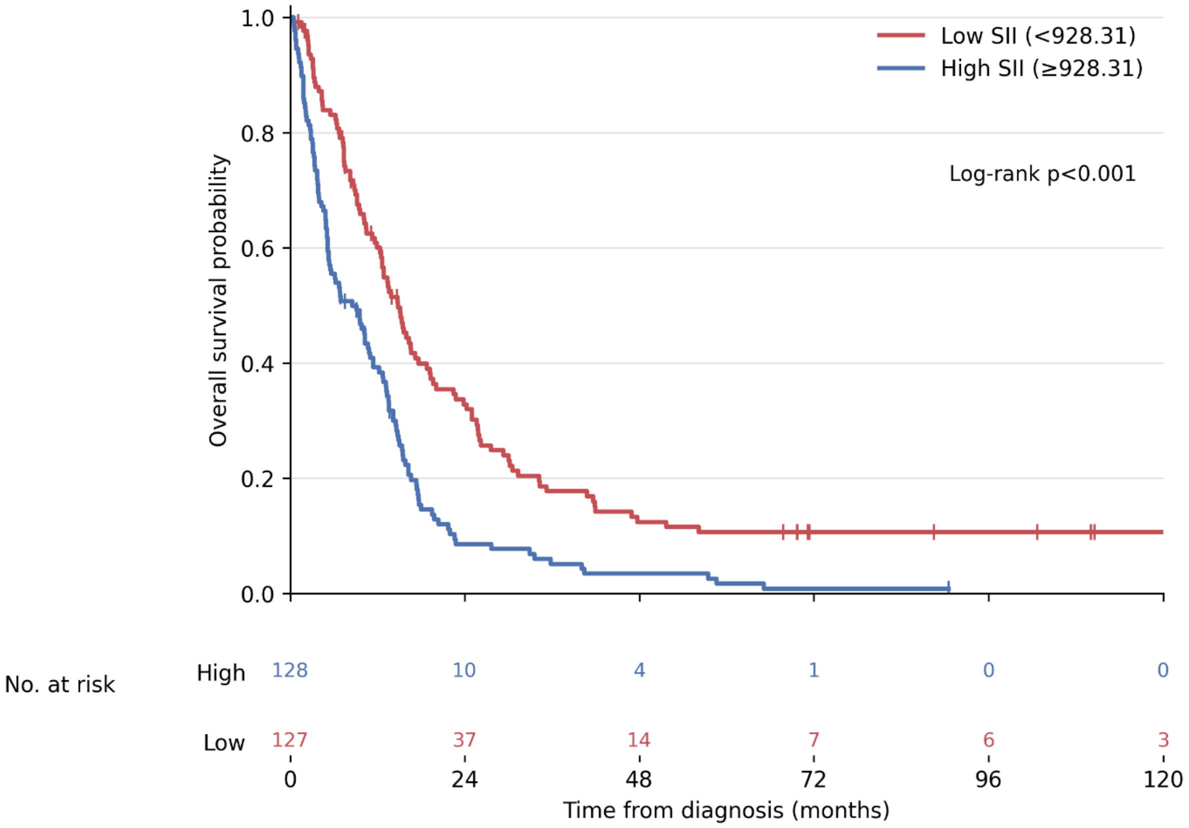

Figure S2. Kaplan–Meier estimates of overall survival according to the systemic immune-inflammation index. Patients were categorized using the cohort median SII value of 928.31. Median overall survival was 14.85 months (95% CI, 12.39–17.18) in the low-SII group and 8.54 months (95% CI, 5.29–11.01) in the high-SII group. Overall survival differed significantly between the groups (log-rank $p<0.001$). Vertical marks indicate censored observations, and the numbers below the plot show patients remaining at risk.
